# Clinical benefit and cost-effectiveness analysis of liquid biopsy application in patients with advanced non-small cell lung cancer (NSCLC): a modelling approach

**DOI:** 10.1007/s00432-022-04034-w

**Published:** 2022-05-09

**Authors:** Fabienne Englmeier, Annalen Bleckmann, Wolfgang Brückl, Frank Griesinger, Annette Fleitz, Klaus Nagels

**Affiliations:** 1 Chair of Healthcare Management and Health Services Research, University of Bayreuth, Parsifalstraße 25, 95445 Bayreuth, Germany; 2 Medical Clinic A, Haematology, Haemostaseology, Oncology and Pulmonology, University Hospital Münster, Albert-Schweitzer-Campus 1, 48149 Münster, Germany; 3 Department of Haematology and Medical Oncology, University of Medicine Goettingen, Robert-Koch-Straße 40, 37075 Göttingen, Germany; 4 Department of Respiratory Medicine, Allergology and Sleep Medicine, Nuremberg Lung Cancer Center, General Hospital Nuremberg, Paracelsus Medical University, Prof.-Ernst-Nathan-Straße 1, 90419 Nuremberg, Germany; 5 Pius-Hospital Oldenburg, University Clinic Internal Medicine, Georgstraße 12, 26121 Oldenburg, Germany; 6 Clinical Epidemiology and Health Economics, iOMEDICO, Ellen-Gottlieb-Straße 19, 79108 Freiburg, Germany

**Keywords:** Liquid biopsy, Cost-effectiveness, NSCLC, Molecular profiling

## Abstract

**Purpose:**

Targeted therapies are effective therapeutic approaches in advanced stages of NSCLC and require precise molecular profiling to identify oncogenic drivers. Differential diagnosis on a molecular level contributes to clinical decision making. Liquid biopsy (LB) use has demonstrated its potential to serve as an alternative to tissue biopsy (TB) particularly in cases where tissue sampling is not feasible or insufficient. We aimed at evaluating the cost-effectiveness of ctDNA-based LB use (molecular multigene testing) according to German care guidelines for metastatic NSCLC.

**Methods:**

A Markov model was developed to compare the costs and clinical benefits associated with the use of LB as an add-on to TB according to the guidelines for NSCLC patients. Usual care TB served as comparator. A microsimulation model was used to simulate a cohort of non-squamous NSCLC patients stage IV. The parameters used for modelling were obtained from the literature and from the prospective German CRISP registry (“Clinical Research platform Into molecular testing, treatment, and outcome of non-Small cell lung carcinoma Patients”). For each pathway, average direct medical costs, and QALYs gained per patient were used for calculating incremental cost-effectiveness ratios (ICER).

**Results:**

The use of LB as an add-on was costlier (€144,981 vs. €144,587) but more effective measured in QALYs (1.20 vs. 1.19) for the care pathway of NSCLC patients (ICER €53,909/QALY). Cost-effectiveness was shown for EGFR-mutated patients (ICER €-13,247/QALY).

**Conclusion:**

Including LB as an add-on into the care pathway of advanced NSCLC has positive clinical effects in terms of QALYs accompanied by a moderate cost-effectiveness.

**Supplementary Information:**

The online version contains supplementary material available at 10.1007/s00432-022-04034-w.

## Introduction

Lung cancer is one of the leading causes of cancer deaths worldwide, with non-small cell lung cancer (NSCLC) being one of the most common entities (Molina et al. 2008; Torre et al. 2015). Histological classified subtypes of NSCLC account for more than 80% of all lung cancer cases including adenocarcinoma, squamous cell carcinoma, large cell carcinoma and carcinomas that are not otherwise specified (NOS). Adenocarcinomas account for about half of these cases (Kraywinkel and Schönfeld 2018). Approximately 75% of patients with NSCLC are diagnosed in an advanced stage and still have a poor diagnosis (Walters et al. 2013). Steadily growing insights into molecular tumour biology and their clinical use in precision oncology are increasingly improving clinical outcomes. The development of targeted therapies has expanded and transformed the therapeutic strategies from conventional modalities such as chemo- and radiotherapy to molecularly targeted therapies. Correspondingly, evolving diagnostic approaches allow physicians to monitor the heterogeneity and evolution of clonally expanded mutations in NSCLC patients. In that respect, activating mutations in the epidermal growth factor receptor (EGFR) represent one of the best known and most commonly found oncogenic drivers in NSCLC, which can be inhibited by targeted therapies (Lung Cancer Group Cologne 2018; Nguyen-Ngoc et al. 2017). The discovery and validation of further genetic alterations have advanced the development of targeted therapies. Among these targets are translocations of anaplastic lymphoma kinase (ALK), translocations of ROS proto-oncogene 1 (ROS1), activating mutations of B-Raf proto-oncogene, serine/threonine kinase in codon 600 (BRAF-V600), and neurotrophic tyrosine receptor kinase (NTRK) gene fusions (Collisson et al. 2014; Farago and Azzoli 2017; Leipert et al. 2019). To initiate a targeted therapy, a molecular pathological examination is inevitable. In that respect, a tissue biopsy (TB) is still considered the gold standard. This diagnostic approach is invasive and limited in examining the heterogeneous, dynamic, and evolving character of a tumour. Furthermore, in clinical practice, several factors can limit the use of TB: The amount of tumour tissue obtained may be too sparse or the tumour cells sampled may be insufficient for molecular testing. In addition, a TB may not be performed due to the poor condition of the patient (Arcila et al. 2011; Chouaid et al. 2014; Dietel et al. 2016; Douillard et al. 2014; Lim et al. 2015). In these cases, a blood-based and minimally invasive liquid biopsy (LB) can be considered as an emerging alternative to identify oncogenic drivers and support clinical decision making (MANDEL and METAIS 1948; Schwartzberg et al. 2020). However, the low concentration of tumour-derived DNA in plasma is yet the major hurdle in LB and requires very sensitive methods (Cheung et al. 2018). Once identified, the molecular diagnostic profile of NSCLC patients can be matched with an appropriate targeted therapy. In Germany, LB is neither part of the standard care nor broadly reimbursed. The primary objective of this study was to evaluate the comparative cost-effectiveness (incremental cost-effectiveness ratio (ICER)) when using LB (ctDNA detection) according to German care pathway guidelines for metastatic non-squamous NSCLC patients as an add-on to TB. The aim was to elucidate the value of LB as a diagnostic procedure in NSCLC patients using the German cancer care setting as a sample.

## Materials and methods

### Population and clinical pathways

The model cohort is characterized by patients with confirmed metastatic non-squamous NSCLC (stage IV). The base case population was divided into five different subgroups depending on their biomarker profiles. We assumed that 13% of patients with non-squamous NSCLC had an EGFR mutation, 2% a BRAF-V600 mutation, 2% an ALK translocation, 1% a ROS1 translocation, and 82% were determined as wildtype/others (Lung Cancer Group Cologne 2018). For all those, except wildtype, at least one approved targeted therapy was available. A TB for pathohistological differentiation has already been performed. Two care pathways were established: one pathway with LB as an add-on to TB (intervention) and a pathway utilizing TB only (comparator). Both diagnostic approaches (TB and LB) as well as the therapeutic regimens were selected based on the German evidence-based care pathway guidelines (Arbeitsgemeinschaft der Wissenschaftlichen Medizinischen Fachgesellschaften e. V. 2018; Griesinger et al. 2019). Real-world data were derived from the CRISP report 2020 (AIO and iOMedico 2021), and consultations with clinical experts (oncologists/co-authors) were performed to confirm clinical relevance. In cases where several therapy options exist for first-line treatment, the most frequently chosen regimen was selected for modelling (chemotherapy and immunotherapy). For targeted therapies, the two most frequently chosen options were evaluated. The frequency of the different therapy sets were obtained from the German CRISP registry (AIO and iOMedico 2021). In addition, clinical experts were consulted for the selection of the most appropriate second-line. The LB is used for detecting ctDNA in plasma to initiate a matched first-line treatment and to identify resistance mutations in the EGFR gene to proceed to treatment change. According to the German guidelines (Arbeitsgemeinschaft der Wissenschaftlichen Medizinischen Fachgesellschaften e. V. 2018; Griesinger et al. 2019), a LB was used when:

- sampled tumour tissue is insufficient for molecular analysis,
- a required TB cannot be performed or
- negative TB (circumstance suggest that findings could be false negative) when testing for resistances for EGFR-TKI.

For modelling purposes, we considered that a histological examination and an immunohistochemical determination of PD-L1 expression had already been performed based on formalin-fixed paraffin-embedded (FFPE) tissue assessment, but a molecular analysis has not yet been carried out. This step represents the starting point of modelling to compare molecular analysis based on TB (comparator) and LB (intervention). If possible, the tissue underwent a molecular pathological examination and was subjected to DNA sequencing for the detection of somatic alterations. DNA sequencing was performed using a targeted next-generation sequencing (NGS) panel. Once the patients had been assigned to the specific oncogenic driver, they received a corresponding matched first-line therapy. Patients were treated until their disease progressed, at which point they were offered another treatment line. In our cohort, a patient could receive a maximum of four treatment lines. All pharmaceuticals included in the model were approved in Germany by June 2019.

### Model overview

**Fig. 1:**
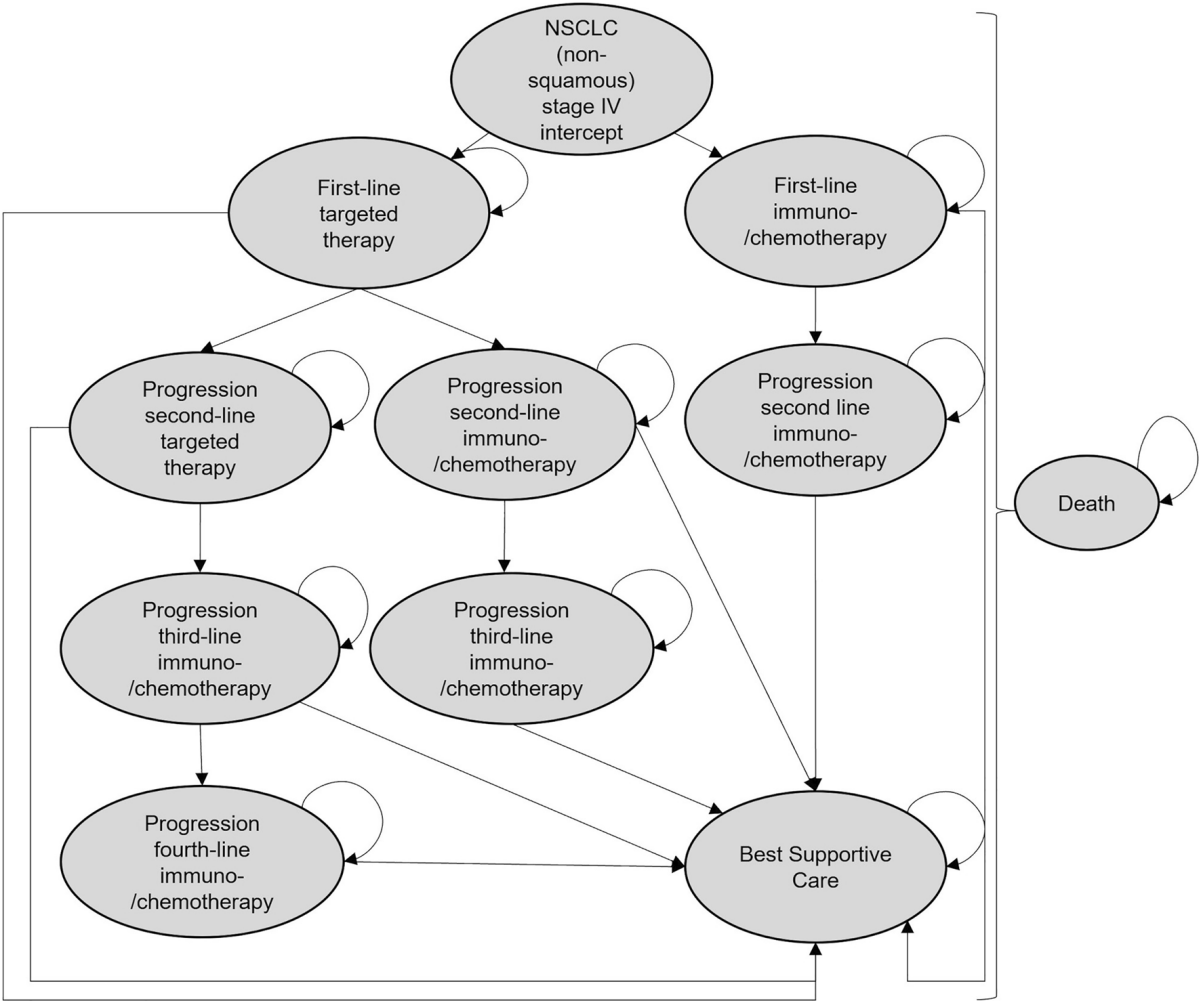
Markov model and its health states used for economic modelling

A Markov model was combined with a decision tree to compare the costs and clinical benefits associated with the use of LB as an add-on to TB according to German care pathway guidelines for non-squamous NSCLC patients. Parts of the modelling were adapted from the health technology assessment of Ontario Health (2020) examine LB to detect EGFR T790M in advanced NSCLC. Our model describes NSCLC (non-squamous) progression over 10 years (120 months) and is based on clinical data derived from literature and a prospective NSCLC registry (CRISP Supplementary Information). CRISP has been established in 2015 and comprises more than 7000 data sets, as of July 2021 (Griesinger et al. 2021). To ensure methodological strength, the guidelines reported in the ISPOR-SMDM Task Force (Caro et al. 2012) were used. Usual care tissue-based diagnosis served as a comparator. The individuals of the Markov cohort could be in different health states (Fig. 1):

- NSCLC intercept: tissue or liquid underwent molecular analysis to determine driver alterations
- First-line treatment/continue to receive treatment
- Progression was noted and next treatment line was initiated
- Progression was noted and best supportive care (BSC) was offered
- Death

The Markov cycle length was expressed in months. In the modelling, a maximum of four lines of therapy were represented, with the simulation terminating in the BSC or death state. It was assumed that after second- and third-line progression 50% of the individuals received BSC (Valdes et al. 2016) and the remaining patients got another line of therapy or died. The proportion who received a second-line after progression could be estimated from the CRISP report. The use of a Markov model is limited by the fact that transition probabilities do not depend on history. In our modelling, different subgroups and treatment lines/regimen were considered, resulting in a vast number of health states increasing the complexity of the model. To circumvent this problem, so-called “trackers” were used. This allowed us to consider which medications the patient received during his or her pathway and how long a patient had already been in a state to adjust adequate transition probabilities.

**Fig. 2:**
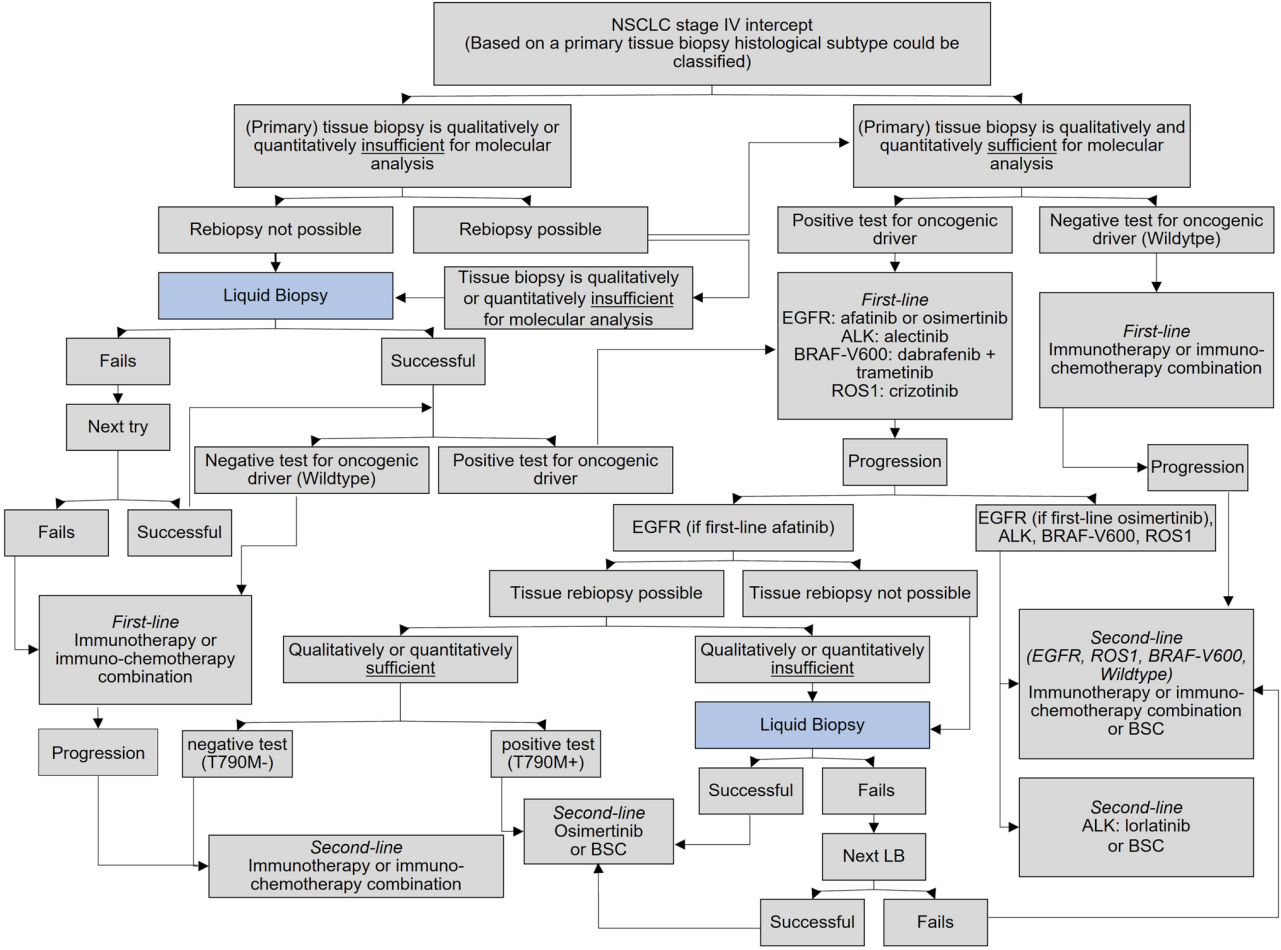
Model structure—Care pathway with liquid biopsy as an add-on**.** Schematic diagram shows the decision tree model structure. It illustrates the care pathway with LB as an add-on and the respective biopsy procedures. *LB* liquid biopsy, *TB* tissue biopsy. Alterations are divided into four gene-subgroups: ALK translocation, EGFR mutations, BRAF-V600 mutation, ROS1 translocation. No oncogenic driver includes wildtypes and other alterations that do not belong to the listed alterations of the genes ALK, BRAF, EGFR, and ROS1. Following agents were used for treatment: EGFR: afatinib (1st-line) – osimertinib (2nd-line); EGFR: osimertinib (1st-line) – atezolizumab + paclitaxel + carboplatin + bevacizumab (2nd-line) or pembrolizumab (2nd-line) or pembrolizumab + pemetrexed + carboplatin (2nd-line); ALK: alectinib (1st-line) – lorlatinib (2nd-line); BRAF-V600: dabrafenib + trametinib (1st-line) – pembrolizumab or pembrolizumab + pemetrexed + carboplatin (2nd-line); ROS1: crizotinib (1st-line) – pembrolizumab or pembrolizumab + pemetrexed + carboplatin (2nd-line); Wildtype and others: pembrolizumab (1st-line) – paclitaxel + carboplatin + bevacizumab (2nd-line); Wildtype: pembrolizumab + pemetrexed + carboplatin (1st-line) – docetaxel + nintedanib (2nd-line)

A decision tree was used to model the different methods of biopsies and molecular testing to initiate the appropriate treatment. Figure 2 shows the care pathway (intervention) with LB as an add-on. The comparator differs in that no LB was offered. Thus, as soon as no molecular analysis could be performed due to qualitatively or quantitatively insufficient tissue, immuno-monotherapy or an immuno-chemotherapy combination was initiated. If progression occurred during the second-line, no further biopsies were performed, which is why the third- and fourth-lines are not shown in Fig. 2. All treatment lines are depicted in Fig. S.1 (Supplementary Information). If there were no more molecular stratified therapies available, combinations of chemo- and/or immunotherapies were initiated depending on PD-L1 expression status.

Our cost-effectiveness analysis adhered to the Consolidated Health Economic Evaluation Reporting Standards (CHEERS) criteria (Husereau et al. 2013). Uncertainty was evaluated through a Monte Carlo Simulation. A microsimulation (10,000 trials) was carried out to depict parameter distributions for diagnostic sensitivity of LB, proportions of second-line treatments, and survival times (PFS and OS). The model was created from the perspective of the statutory health insurance (SHI). In addition to the direct medical costs relevant to the SHI, co-payments incurred by the insured for medication were also considered. We used a time horizon of 120 cycles.

### Outcomes

The primary clinical outcomes and related resource consumption in the model were measured in terms of QALYs and direct medical cost associated with the care pathway defined above. Mean PFS and OS were also reported. Microsimulation was used to calculate the mean values of gained QALYs, direct medical cost and the ICER. The ICER represents the average incremental cost associated with one additional QALY and represents the economic value of an intervention, compared with a comparator.

$${\text{ICER}} = \frac{{{\text{Cost Intervention}} - {\text{Cost Comparator}}}}{{{\text{QALY Intervention}} - {\text{QALY Comparator}}}}$$

Costs were discounted at an annual rate of 3% as the time horizon for the analysis was more than 1 year, in line with German guidelines of the IQWiG (Institut für Qualität und Wirtschaftlichkeit im Gesundheitswesen 2019). All costs were stated in Euro (data from 2020). No cost-effectiveness threshold was set (willingness-to-pay of 0 €) since no maximum cost per health outcome (QALY) has been defined in the German health system setting.

### Survival times and transition parameters

The parameters used for modelling were obtained from published clinical trials, clinical registry data (AIO and iOMedico 2021) or**,** where appropriate, assumptions were derived by clinical expert opinion. The compilation of the median PFS and OS data (estimated by Kaplan–Meier analysis) related to the different therapy lines were carried out through a systematic literature search completed in June 2020 (see Supplementary Data for more information).

We extracted OS and PFS data (Table S.2 Supplementary Information) to determine the transition probabilities between the health states. The freely accessible platform WebPlotDigitizer (Rohatgi 2021) was used to digitize the Kaplan–Meier curves. Subsequently, the Kaplan–Meier curves could be reconstructed with the help of the statistical software R (version 4.0.4) according to the procedure described by Guyot et al. 2012. The packages "MASS" (Venables and Ripley 2002), "splines" (Wang and Yan 2021a, b) and "survival" (Therneau 2021; Therneau and Grambsch 2000) were used. The obtained data could be checked for various distributions in R using the package "flexsurv" (Jackson 2016). At the time of the literature search, there were no robust survival data for lorlatinib in the second-line treatment for ALK translocations. Based on a subsequent research (as of 15.01.2022), the study of Frost et al. (2021) was identified. In this study, Kaplan–Meier curves were depicted, which made an estimate of distributions possible. The selected distributions for the respective survival data are listed in Table 1.

**Table 1:** Distributions used for overall and progression-free survival

Distribution	Name	Shape/mean of logs	Scale/SD of logs/rate	*k* (kappa)
GeneralizedGamma	OS afatinib	0.6540	3.0200	4.27
LogNormal	OS alectinib	4.6060	2.0840	
GeneralizedGamma	OS atezolizumab, bevacizumab, carboplatin, paclitaxel	0.2570	0.0014	11.34
LogNormal	OS Best Supportive Care	2.4235	0.9265	
Weibull	OS bevacizumab, carboplatin, paclitaxel	1.6180	22.4480	
LogNormal	OS crizotinib	3.9010	1.6110	
LogNormal	OS dabrafenib, trametinib	3.0800	0.9870	
GeneralizedGamma	OS docetaxel, nintedanib	0.3290	0.0169	9.13
LogNormal	OS lorlatinib	2.8570	1.5900	
GeneralizedGamma	OS osimertinib (first-line)	2.1590	51.6980	0.757
LogLogistic	OS osimertinib (second-line	1.9700	27.0200	
LogNormal	OS pembrolizumab (first-line)	3.1932	0.6839	
LogNormal	OS pembrolizumab (second-line)	2.7739	0.7366	
GeneralizedGamma	OS pembrolizumab, carboplatin, pemetrexed (PD-L1 < 1%)	0.6240	4.8200	2.58
GeneralizedGamma	OS pembrolizumab, parboplatin, pemetrexed (PD-L1 1–49%)	0.7310	10.0100	2.08
LogLogistic	PFS afatinib	2.0090	11.0180	
LogNormal	PFS alectinib	3.4180	1.8340	
LogNormal	PFS atezolizumab, bevacizumab, carboplatin, paclitaxel	2.2940	0.8820	
LogLogistic	PFS bevacizumab, carboplatin, paclitaxel	2.3890	7.0480	
LogNormal	PFS crizotinib	3.0140	1.4130	
GeneralizedGamma	PFS dabrafenib, trametinib	0.5230	0.5260	5.31
LogNormal	PFS docetaxel, nintedanib	1.2984	0.8088	
LogNormal	PFS lorlatinib	2.0400	1.1950	
LogNormal	PFS osimertinib (first-line)	2.8872	0.8694	
LogNormal	PFS osimertinib (second-line)	2.3090	0.9369	
LogNormal	PFS pembrolizumab (first-line)	2.0541	1.2435	
LogNormal	PFS pembrolizumab (second-line)	1.6212	1.3639	
LogNormal	PFS pembrolizumab, carboplatin, pemetrexed (PD-L1 < 1%)	1.8826	1.0248	
GeneralizedGamma	PFS pembrolizumab, carboplatin, pemetrexed (PD-L1 1–49%)	0.4720	0.4170	4.88

The diagnostic accuracy of TB and LB was obtained from technical data of companion diagnostics (Clark et al. 2018; Food and Drug Administration 2020; Foundation Medicine 2020). To determine sensitivity for LB, we assumed a mutant allele frequency (MAF) for our cohort between 0.25 and 0.5%, which is consistent with published results of a median MAF of 0.43% by Mack et al. (2020). Since limited data availability for the sensitivity of LB, triangular distributions were used considering the minimum, maximum and the peak of the given confidence intervals (CI). The PPV was set at 100% for both tissue and LB following Clark et al. (2018), whose analytical validation results were approximately 100%.

### Utilities

To determine the ICER, QALYs were used as utility values. These are generally derived from two dimensions—remaining life expectancy and quality of life. The quality of life can take on values between 0 and 1, whereby the value 1 can be understood as complete health without any impairments, whereas a QALY of 0 corresponds to death. The QALYs were obtained by multiplying the quality of life by the remaining lifetime. The modelling considered the impact of treatment lines and their side effects as well as complications during bronchoscopy (pneumothorax 2%, Ost et al. 2016) on quality of life. To assess these parameters, the publications of Nafees et al. (2008), Nafees et al. (2017), Chouaid et al. (2013) and Handorf et al. (2012) were used. To determine the quality of life during a line of therapy, different utilities were estimated for several health states (progression, stable disease, response). Subsequently, the frequency of relevant side effects (diarrhoea, fatigue, febrile neutropenia, hair loss, nausea/vomiting, neutropenia, rash, bleeding, and hypertension) were extracted from clinical trials identified by our literature research. Only serious adverse events (grade ≥ 3) were considered. Utility decrements of side effects were taken from the publication of Nafees et al. (2017) and the utility decrements of a pneumothorax (reduced utility of − 0.04) could be obtained from Handorf et al. (2012) (see also Table S.3, Table S.4, and Table S.5 Supplementary Information).

### Costs

The health economic analysis considered the setting of the German healthcare system and entails the following direct medical costs associated with NSCLC treatment: drug costs, diagnostic costs, and expenses for molecular pathological examinations. To estimate the unit cost of drugs, the drug dosages were taken from the Summary of Product Characteristics (SmPC). The calculated dosage for patients is based on the average height (1.72 m) and weight (77 kg) of an adult in Germany (Statistisches Bundesamt 2018) resulting in a body surface area of 1.90 m^2^. In accordance with the German “Lauer-Taxe” and Hilfstaxe (as of 17.06.2020), the latest drug prices including co-payments of insured patients had been determined. To estimate the annual therapy costs, the treatment mode, the number of treatments per patient per year, the treatment duration per treatment in days and the resulting treatment days per year were determined. Details are provided in Table S.6 (Supplementary Information). LB incurs costs for blood sampling and molecular laboratory, while a TB requires complex invasive methods and pathological assessment. We assumed a bronchoscopy along with biopsy sampling as a standard workup for NSCLC patients. Tissue collection can be performed on an outpatient or inpatient basis. For inpatient TB, the corresponding OPS (Operationen- und Prozedurenschlüssel) codes, and the ICD-10 code C34 were used to calculate the respective G-DRG (German Diagnosis Related Groups). We assume a stay of two days in hospital for inpatient TB and one ambulatory visit. Cost data for outpatient biopsies were estimated using the German physician fee schedule and catalogue (Einheitlicher Bewertungsmaßstab, EBM), respectively. Costs of the pathological examination could also be determined based on the fee schedule codes. Further input parameters used for modelling are depicted in Table S.7 (Supplementary Information).

### Statistical analysis

The software TreeAge Pro Healthcare Version 2020 R1.2 (TreeAge Pro 2021) was used for modelling and analyses. Microsimulation with 10,000 trials were used and carried out for different subgroups:

- Total cohort (driver alterations and wildtype/others)
- ALK-translocated patients
- BRAF-V600-mutated patients
- EGFR-mutated patients
- ROS1-translocated patients
- Driver alterations (comprising patients with ALK, BRAF-V600, EGFR and ROS1 alterations)

The base case cohort (cohort I) consists of patients with predefined probabilities if sampled tumour tissue is insufficient for molecular analysis or a required tissue rebiopsy cannot be performed. Separate calculations were conducted for the sub-cohort (cohort II) once again, in whom sampled tumour tissue is insufficient for molecular analysis or a required tissue rebiopsy cannot be performed. One-way sensitivity analyses were conducted to determine key drivers of outcomes. The results of several univariate analyses are presented in a tornado diagram (Fig. 6). Plausible ranges of the selected variables were used, and each variable was tested at the upper and lower limits correspondingly. To depict a cost-effectiveness acceptability curve, a probabilistic sensitivity analysis (PSA) was carried out to show the probability that a care pathway is cost-effective at various willingness-to-pay (WTP) values.

## Results

**Fig. 3:**
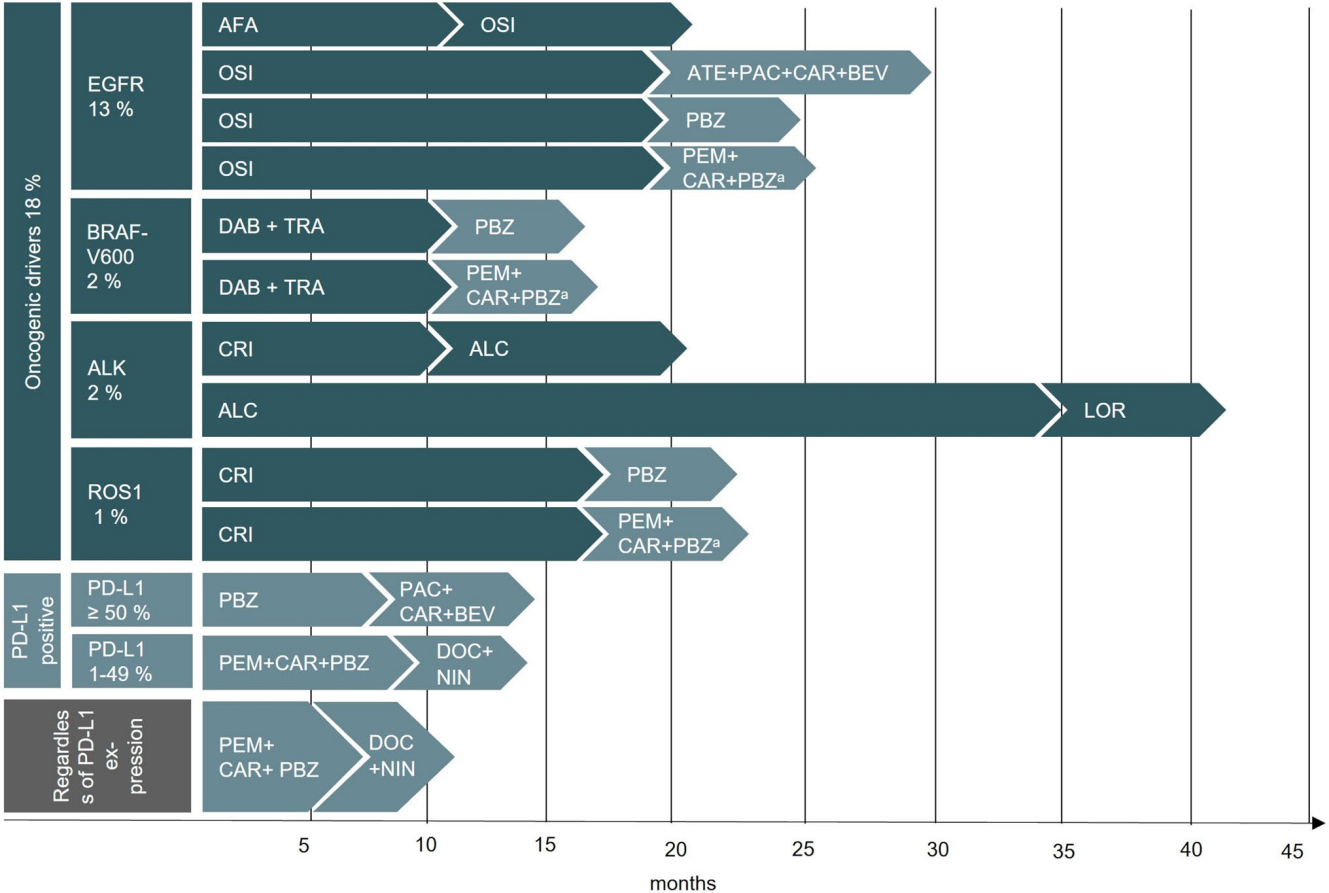
Treatment lines of non-squamous NSCLC and corresponding progression-free survival. Illustration of the current personalized treatment options for non-squamous NSCLC. The therapies were selected according to evidence-based German guidelines. If more than two therapies were available for first- or second-line use the two most frequently prescribed substances were selected according to the German CRISP report 2020. ^a^Since the combination of PEM + CAR + PBZ is currently under investigation in the second-line setting (KEYNOTE-789 (U. S. National Library of Medicine/ClinicalTrials.gov 2018)), the clinical parameters for the combination of PAC + CAR + BEV were used for modelling purposes. *AFA* afatinib, *ALC* alectinib, *ATE* atezolizumab, *BEV* bevacizumab, *CAR* carboplatin, *CRI* crizotinib, *DAB* dabrafenib, *DOC* docetaxel, *LOR* lorlatinib, *NIN* nintedanib, *OSI* osimertinib, *PAC* paclitaxel, *PBZ* pembrolizumab, *PEM* pemetrexed, *TRA* trametinib

**Fig. 4:**
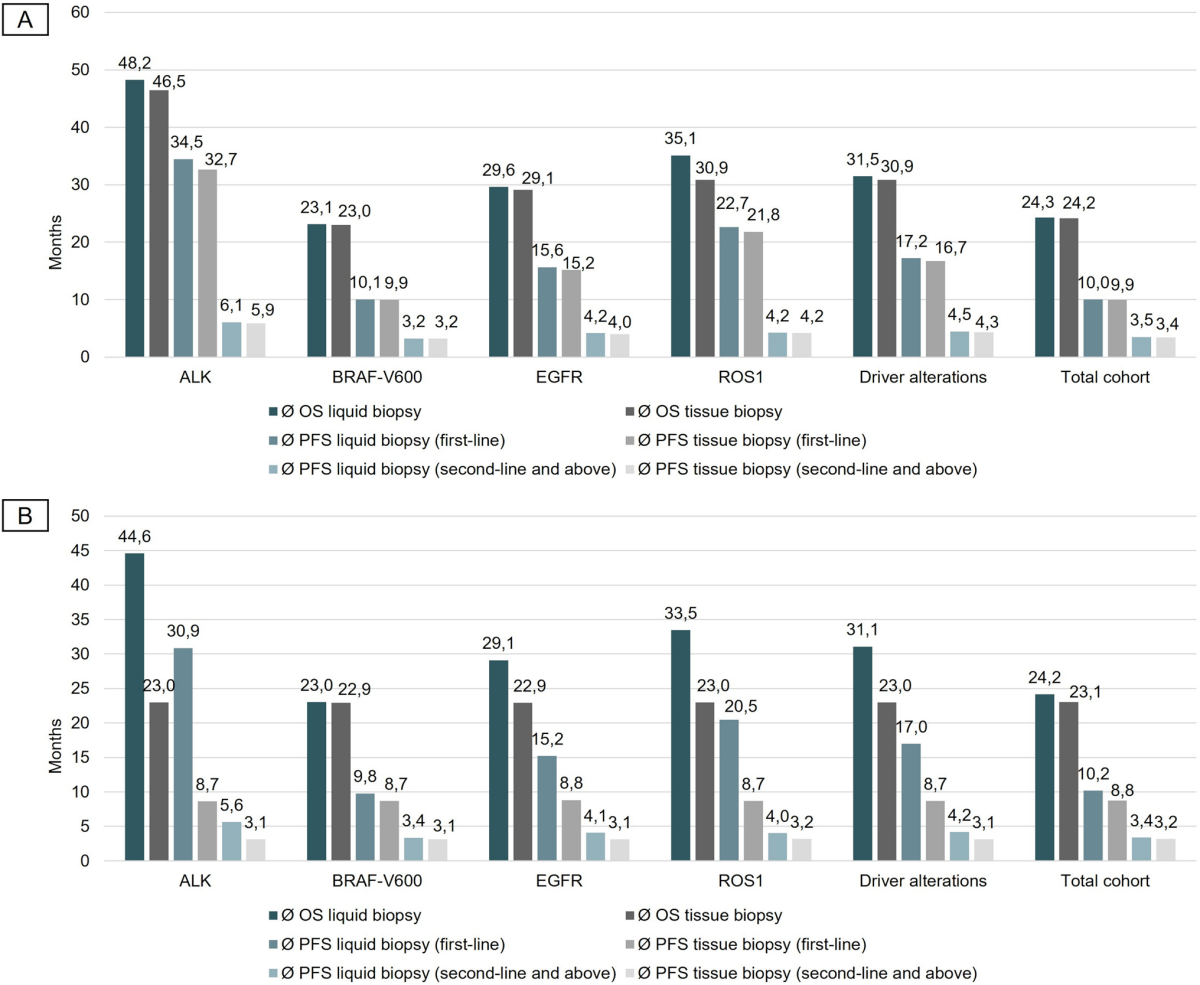
Progression-free survival (PFS) and overall survival (OS) derived from the respective therapy lines of the corresponding care pathways. **A** Subgroup (cohort I) includes all patients, regardless of whether a tissue biopsy can be taken, or the tissue is sufficient for molecular analysis. **B** Subgroup (cohort II) includes patients in whom tissue rebiopsy or molecular analysis on primary tissue sample is not possible. The figure indicates the survival data achieved in the respective care pathway. The survival data were calculated for different subgroups. Driver alterations are divided into four subgroups: ALK translocation, EGFR mutations, BRAF-V600 mutation, ROS1 translocation; The total cohort includes all NSCLC cases (wildtype, and driver alterations of the genes ALK, BRAF, EGFR, and ROS1)

The median PFS and median OS of respective therapies were taken from 29 studies covering first-line treatment regimens or above (Fig. S.2 and Table S.2 Supplementary Information). As there were no reliable survival data for alectinib in second-line ALK-translocated patients, alectinib was used exclusively as first-line in the modelling (second-line lorlatinib). The survival data of immuno-chemotherapy combinations that were used beyond the first-line could not be explicitly assigned to a therapy-line based on the clinical studies. Therefore, the survival data were assumed for the second-line as well as for the lines of treatment beyond. The longest attainable median PFS with 34.8 months (95% CI 17.7-not estimable) could be achieved by alectinib for treatment of ALK-translocated patients. If no molecular testing was feasible due to insufficient tissue, first-line combination-therapy (chemotherapy and immunotherapy) or immune-monotherapy and second-line combination-therapy (chemotherapy and immunotherapy) were assumed as chosen approach, resulting in a median PFS for first-line of 6.2–9.2 months and second-line or above of 4.2–6.1 months. The median PFS for different treatments are depicted in Fig. 3. The results of the microsimulation for cohort I demonstrated that the use of a LB as an add-on was associated with an extended mean PFS (10.0 months vs. 9.9 months) in first-line and a prolonged mean OS (24.3 months vs. 24.2 months) in total cohort and in all subgroups (Fig. 4A). For ALK-translocated patients, the mean PFS in first-line with a LB was 34.5 months [95% CI 33.7–35.2] and without LB 32.7 months [95% CI 31.9–33.4]. A mean OS of 48.2 months [95% CI 47.5–49.0] vs. 46.5 months [95% CI 45.7–47.2] was observed. The least advantage of a LB for first-line could be gained by the mutation BRAF-V600 (mean PFS 10.1 months [95% CI 9.9–10.2] vs. 9.9 months [95% CI 9.8–10.1]). For EGFR and ROS1 alteration, a mean PFS of 15.6 months [95% CI 15.4–15.9] vs. 15.2 months [95% CI 14.9–15.4] and 22.7 months [95% CI 22.1–23.2] vs. 21.8 months [95% CI 21.3–22.3]) was gained for first-line, respectively.

Moreover, results suggest that patients for whom TB sampling is not feasible or molecular analysis not possible (cohort II) significantly benefit from LB application regarding PFS for first-line treatment and OS (Fig. 4B). The care pathway with LB resulted in a mean PFS of 10.2 months [95% CI 9.9–10.4] and a mean OS of 24.2 months [95% CI 23.8–24.6], whereas offering only TB yielded a mean PFS of 8.8 months [95% CI 8.6–8.9] and a mean OS of 23.1 months [95% CI 22.7–23.5] for all patients with non-squamous NSCLC of cohort II. The highest gain (Δ) in PFS achieved in first-line by offering LB could be observed in patients with ALK translocations, resulting in Δ 22.2 months (EGFR Δ 6.4 months, BRAF-V600 Δ 1.1 months, ROS1 Δ 11.8 months).

**Fig. 5:**
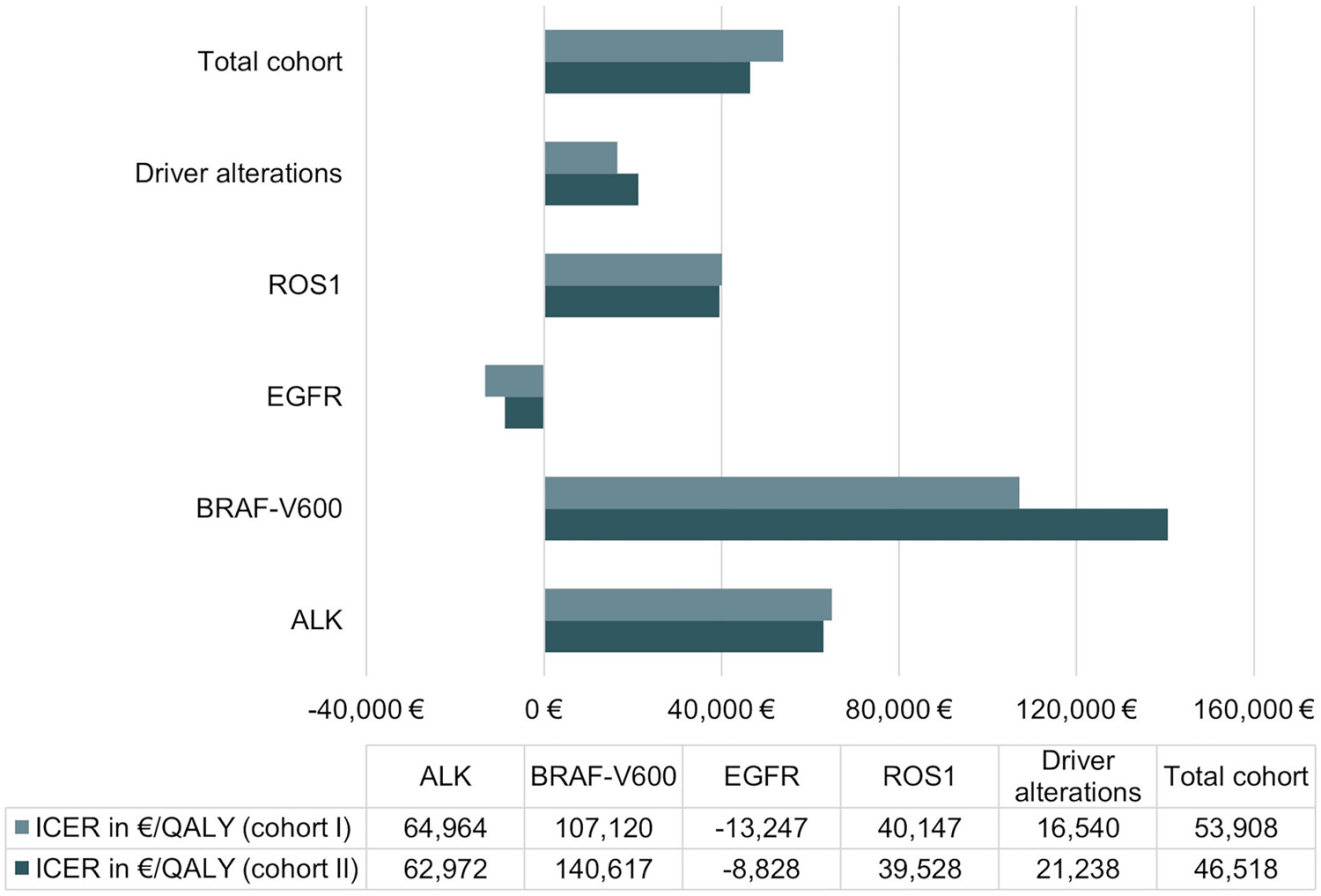
ICER of the competing care pathways. ICER indicates incremental cost-effectiveness ratio for the subgroups based on the underlying biomarker profile. The ICER of the total cohort (cohort I) is presented, and the ICER of the subgroup in which TB or molecular analysis on tissue sample is not possible (cohort II)

For the total cohort (cohort I), the use of LBs as an add-on was costlier (€144,981 [95% CI 142,545–147,417]) but clinically more effective (1.20 QALY [95% CI 1.18–1.21]) than a pathway without LB resulting in an ICER of €53,908/QALY. Focussing on the subgroup with driver alterations an ICER of €16,540/QALY was calculated. A care pathway without LB was associated with direct medical costs of €144,587 [95% CI 142,145–147,029] and resulted in 1.19 QALYs [95% CI 1.17–1.21], shown in Table 2. Considering the costs and QALYs that arise for patients with molecular alterations and corresponding matched therapy, the ICER and cost-effectiveness differed between the subgroups (Table 3). For the EGFR gene, the care pathway with LB showed a negative ICER of €-13,247/QALY in patients with an activated mutation and dominates the pathway without LB for. For the remaining alterations, the use of LB was associated not only with an improved PFS, OS and QALYs but also with higher costs (Table 3). Thus, modelling data strongly suggest that LB application regarding EGFR can both save costs and increase QALYs, resulting in a significant cost-effectiveness favouring LB. The calculated ICER of the care pathway with and without LB as an add-on for different alterations are shown in Fig. 5. Considering the cohort II in whom sampled tumour tissue is insufficient for molecular analysis or a required tissue rebiopsy cannot be performed, the results on the ICER were similar to cohort I.

**Table 2:** QALYs and direct medical costs for the competing care pathways and the corresponding ICER

	Total cohort		Driver alterations	
	Care pathway LB as an add-on	Care pathway TB	Care pathway LB as an add-on	Care pathway TB
Total cost (in €)	144,981 €	144,587 €	152,399 €	151,669 €
95% lower bound	142,545 €	142,145 €	149,910 €	149,168 €
95% upper bound	147,417 €	147,029 €	154,887 €	154,171 €
Drugs	140,212 €	140,100 €	147,446 €	147,016 €
95% lower bound	137,775 €	137,658 €	144,956 €	144,513 €
95% upper bound	142,649 €	142,542 €	149,935 €	149,519 €
Tissue biopsy	4,493 €	4,487 €	4,676 €	4,653 €
95% lower bound	4,450 €	4,445 €	4,632 €	4,610 €
95% upper bound	4,535 €	4,529 €	4,720 €	4,696 €
Liquid biopsy	276 €	0 €	277 €	
95% lower bound	257 €	0 €	258 €	
95% upper bound	296 €	0 €	296 €	
Effectivity QALY	1.20	1.19	1.71	1.67
95% lower bound	1.18	1.17	1.68	1.64
95% upper bound	1.21	1.21	1.74	1.69
Mean PFS (first-line)	10.0	9.9	17.2	16.7
95% lower bound	9.8	9.7	16.9	16.3
95% upper bound	10.2	10.2	17.6	17.1
Mean PFS (second-line and above)	3.5	3.4	4.5	4.3
95% lower bound	3.3	3.2	4.3	4.1
95% upper bound	3.7	3.6	4.6	4.5
Mean OS	24.3	24.2	31.5	30.9
95% lower bound	23.9	23.8	31.0	30.4
95% upper bound	24.7	24.6	32.0	31.3
Incr. Cost	393 €		729 €	
Incr. QALYs	0.01		0.04	
ICER (€/QALY)	53,908 €		16,540 €	

Due to rounding, there may be discrepancies in the totals

*LB* liquid biopsy, *TB* tissue biopsy

**Table 3:** QALYs and direct medical costs for the competing care pathways and the corresponding ICER differentiated according to alterations

	EGFR		ALK	
	Care pathway LB as an add-on	Care pathway TB	Care pathway LB as an add-on	Care pathway TB
Total cost (in €)	136,705 €	137,248 €	247,243 €	239,514 €
95% lower bound	134,764 €	135,265 €	242,985 €	235,330 €
95% upper bound	138,646 €	139,231 €	251,502 €	243,698 €
QALY	1.60	1.56	2.82	2.70
95% lower bound	1.59	1.55	2.77	2.65
95% upper bound	1.62	1.58	2.86	2.74
ICER	− 13,247 €		64,964 €	

	BRAF-V600		ROS1	
	Care pathway LB as an add-on	Care pathway TB	Care pathway LB as an add-on	Care pathway TB
Total cost (in €)	151,333 €	150,290 €	173,933 €	172,082 €
95% lower bound	149,202 €	148,141 €	171,084 €	169,238 €
95% upper bound	153,463 €	152,438 €	176,781 €	174,925 €
QALY	1.19	1.18	1.90	1.86
95% lower bound	1.17	1.16	1.87	1.83
95% upper bound	1.20	1.19	1.94	1.89
ICER	107,120 €		40,147 €	

Due to rounding, there may be discrepancies in the totals

*LB* liquid biopsy, *TB* tissue biopsy

### Sensitivity analysis

**Fig. 6:**
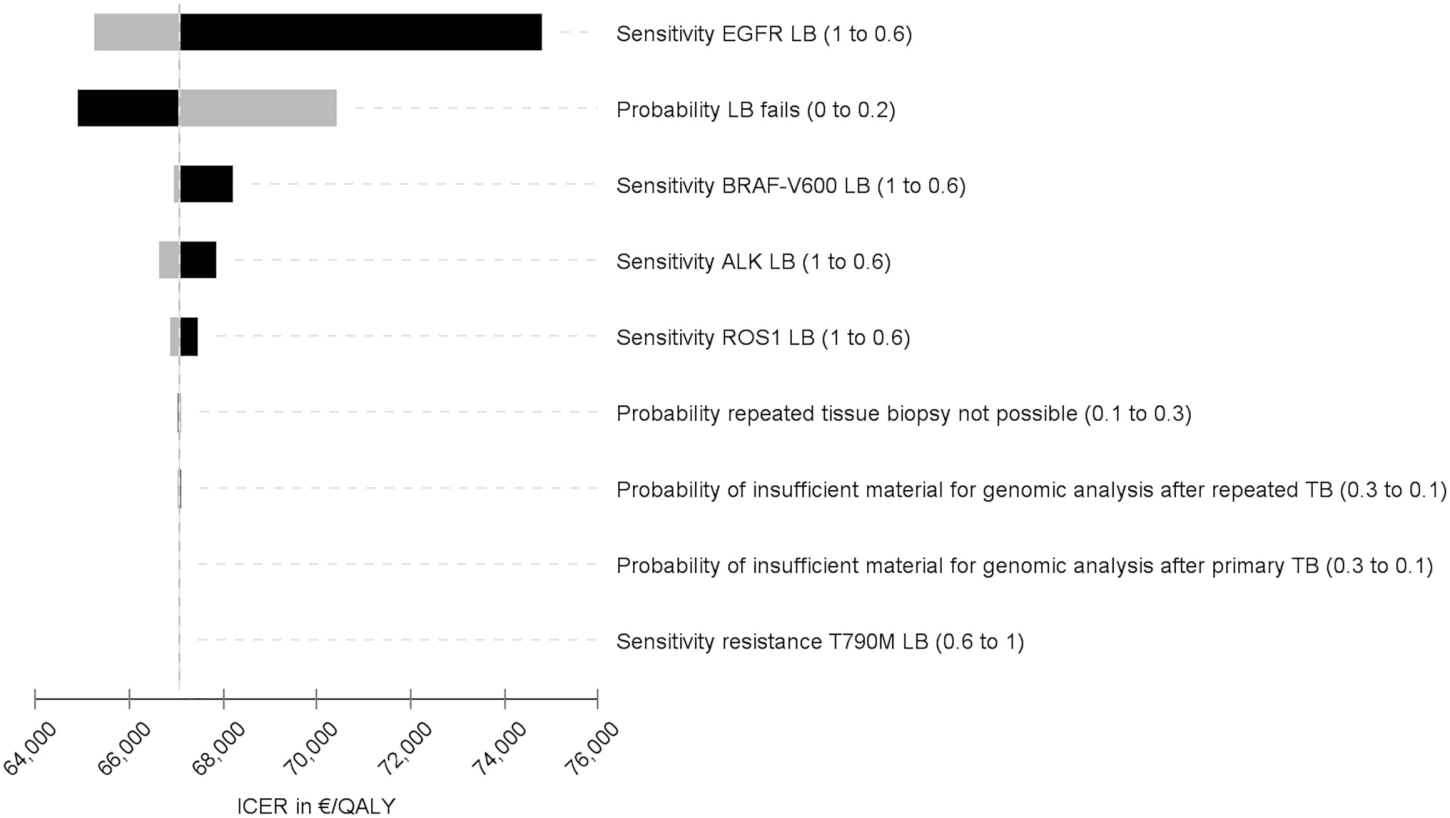
Tornado diagram and the influence on the ICER. The tornado diagram is based on a cost-effectiveness simulation without microsimulation. The expected value (EV) deviates from the EV calculated by the 10,000 trials via microsimulation. Black: low value; grey: high value

**Fig. 7:**
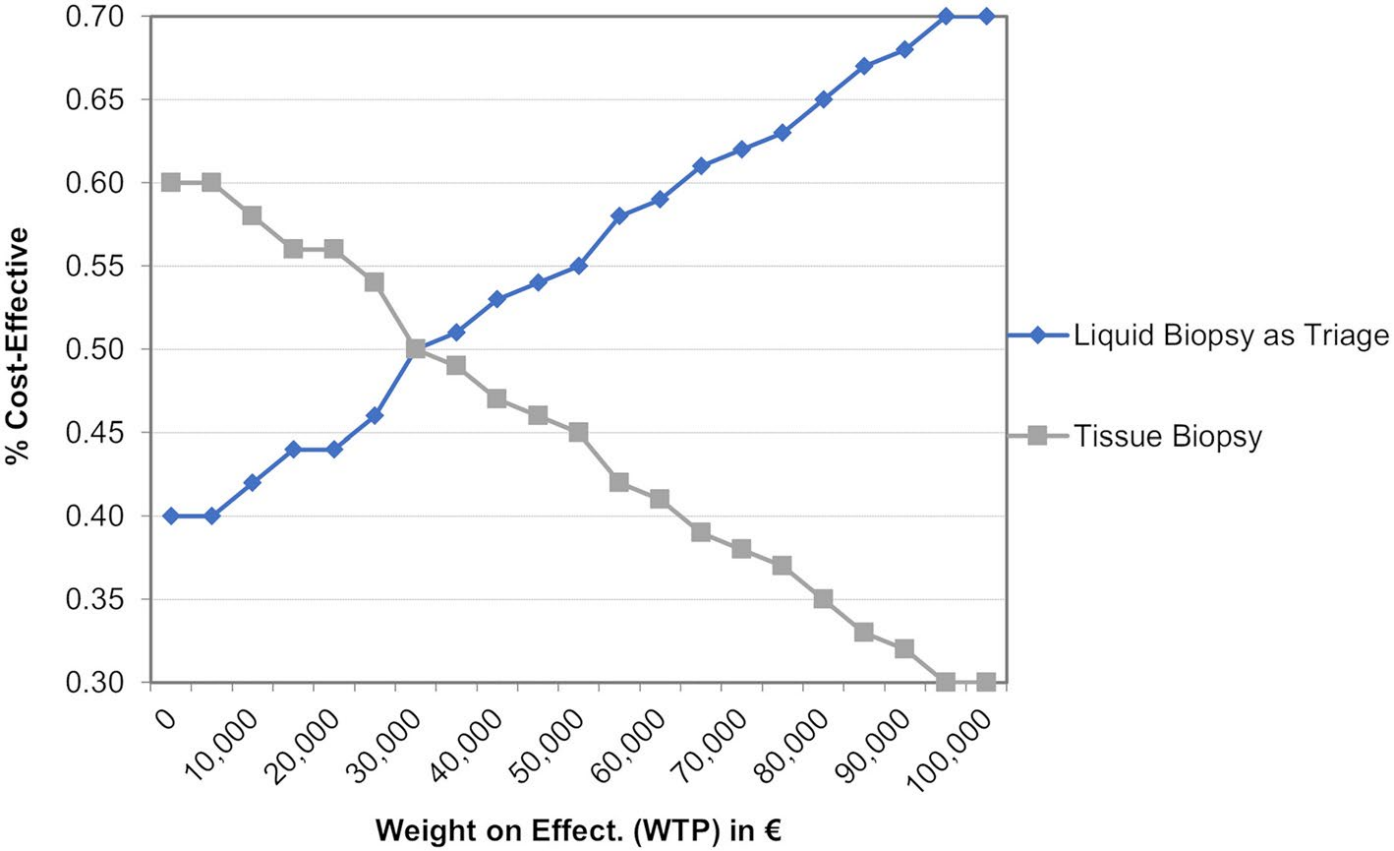
Cost-effectiveness acceptability curve. A curve illustrating the probability that a care pathway is cost-effective based on different WTP values for one gained QALY

The results of the univariate sensitivity analyses (Fig. 6) suggest that sensitivity of LB has significant effects on the ICER. Regarding molecular analysis, the feasibility of tissue and the possibility of a genomic analysis after primary TB affect the ICER. If molecular pathology analysis cannot be performed, targeted therapies would not be used resulting in shorter survival times and lower QALYs, thus favouring a care pathway with LB as an add-on. A loss of PFS and OS instead provides cost savings as therapies are only administered over a shorter time frame. Results from the PSA are depicted in Fig. 7. The figure shows the probability of cost-effectiveness depending on various values for the WTP. At a WTP of €30,000 per QALY or greater the care pathway with LB as an add-on had the highest probability of being cost-effectiveness.

## Discussion

To our knowledge, the current study is the first to evaluate the incremental cost-effectiveness of utilizing a molecular diagnostic testing strategy based on LB as add-on to TB for different underlying alterations in comparison to a diagnostic strategy limited to standalone TB. The potential of LB was demonstrated for following indications: (i) sampled tumour tissue is insufficient for molecular analysis, (ii) a required tissue rebiopsy cannot be performed (iii), and LB used to validate negative test results in case of suspected therapy resistance. In general, the use of LB as an add-on in care pathways for patients diagnosed with metastatic NSCLC had positive clinical effects in terms of PFS, OS and QALY gains. This is due to the increased use of personalized treatments compared to a care pathway with standalone TB. The use suggests a moderate cost-effectiveness, depending on the genetic alteration and the WTP. As far as the total cohort is concerned, there is only a little difference in the total costs and benefit of the care pathways. The small difference in cost should not be a reason for not offering optimal diagnostics and treatment, even if the overall benefit is small. Thus, the widespread use of LB as an add-on should be considered, as the benefit for the subgroups with treatable driver mutations may well be significant (Fig. 4A and B).

LB-based molecular profiling enables clinical decision making that results in the initiation of less expensive molecular targeted therapies as compared to more costly combination therapies (e. g. immune checkpoint-inhibitors plus chemotherapy (pembrolizumab plus carboplatin plus pemetrexed) resulting in treatment cost per year of €171,006 € compared with osimertinib, which costs €70,638. Remon et al. (2020) showed that personalized treatment in advanced NSCLC patients with actionable alterations, detected by LB genomic profiling, achieved a 3-month disease control rate of 86% and a median PFS of 14.8 months irrespective of the chosen therapy line. These data are in line with our data with a mean PFS for first-line of 17.2 months.

In the present modelling, LB contributes to both the precise selection of first-line therapy for patients with treatable driver mutations and the initiation of second-line in EGFR cases with evolving resistance (T790M). The cost-effectiveness of using LB in the care pathway is particularly indicated in patients with EGFR-mutated tumours. This can be explained by the fact that common resistance mechanisms in EGFR are understood in detail and thus appropriate targeted therapies can be initiated. Afatinib in first-line treatment is less costly than mono-immunotherapy (PBZ) or an immuno-chemotherapy (PBZ + CAR + PEM), and if the resistance T790M occurred and can be detected, osimertinib is an effective second-line treatment leading to prolonged survival data and QALYs compared to cytotoxic agents. Analysis of the underlying alterations for EGFR-mutated patients is accepted by payers, which in Germany are dominantly statutory health insurances, and reimbursement is granted accordingly e.g., by the EBM codes 19,460/19461 (Kassenärztliche Bundesvereinigung 2020). Use of LB also appears to be promising for patients with ALK translocations to initiate second-line therapy since resistance mechanisms have also been well understood and targeted therapies are available (Rothenstein and Chooback 2018; Shaw et al. 2019; Solomon et al. 2018).

In addition to targeted therapies, immune checkpoint inhibitor (ICI) therapies have gained importance in the last years and have demonstrated prolonged survival even for NSCLC patients in advanced stages. However, the objective response rate (ORR) remains at 40–50% (Mok et al. 2019; Reck et al. 2016, 2019) for pembrolizumab monotherapy in first-line. Therefore, the overall comparative assessment needs to critically reflect that targeted therapies at least have higher ORR (Peters et al. 2017; Planchard et al. 2017; Shaw et al. 2014; Solomon et al. 2014; Soria et al. 2018; Wu et al. 2014, 2018; Zhou et al. 2019). Additionally, ICI monotherapy is highly ineffective as first-line palliative treatment in patients with EGFR or ALK driver mutations (Miyawaki et al. 2020; Rihawi et al. 2017). Therefore, molecular targeted therapies based on targeting specificity can improve treatment effectiveness, and safety for NSCLC patients (Ai et al. 2018).

In general, taking the value of diagnostic information on oncogenic effects into account, not only NSCLC patients may benefit from LB, but also patients with colorectal (Bettegowda et al. 2014; Siravegna et al. 2015) or breast cancer (Bettegowda et al. 2014) due to a high concordance between TB and LB. Translation of complex diagnostic innovation remains a challenge although the impact on outcome is growing. Health systems handle related issues heterogeneously. As for instance, a molecular analysis based on blood samples is not generally reimbursed throughout all German care settings compared to the health care system in France. Accordingly, this suggests disparate translation of innovation and access to advanced cancer care, particularly in inpatients settings that hinders timely access to highly relevant diagnostic information and can lead to considerable delays in the clinical decision making process (DKG – aktuell 2020). In the outpatient sector patients have an easier access to molecular diagnostics such as LB, e. g. molecular pathology testing using LB for selected EGFR mutations have already been reimbursed in Germany. The national Network Genomic Medicine (nNGM) aims to implement nation-wide comprehensive molecular diagnostics to ensure that all patients with advanced lung cancer in Germany will have access to molecular diagnostics and innovative therapies. But inconsistent and partly incomprehensible reimbursement policies impact health care delivery. Accordingly, this may pose a reason for the fact that despite recommendations of the German care guidelines, not all patients are tested on a regular basis for clinically relevant biomarkers like EGFR, ALK, BRAF or ROS1 (Ostermann et al. 2020).

Our analysis should be interpreted in the context of general limitations. Follow-up studies are needed for some of the clinical studies evaluated to make a more definitive estimate of the survival data. Although the model reflects the actual care situation, it is limited by the fact that age-specific outcome probabilities were not considered to reduce the complexity of modelling. We had only limited access to sensitivity of TB and for LB only data of one manufacturer were used. To obtain a comprehensive analysis into the costs, side effects caused by the therapy lines as well as the complications such as that observed with bronchoscopy may need to be considered in more detail. Furthermore, some biases cannot be fully excluded: i.e., patients in whom no molecular analysis can be performed by tissue may be in a worse clinical state of health as compared to those in whom the molecular analysis could be performed using TB. One limitation of LB is a lower analytical sensitivity compared to TB. However, LB has the advantage of detecting different mutations from different sites of the primary tumour and metastases. When interpreting the results of our modelling, it must be considered that different therapy regimens are used for the alterations and only a restricted number of treatment lines were considered. Deviating treatments may influence QALYs and cost. In addition, it was not possible to consistently identify the therapy lines relevant for the modelling and the corresponding clinical endpoints. The clinical endpoints of a combination of paclitaxel, carboplatin and bevacizumab in the second-line had to be assumed for the combination of pembrolizumab, carboplatin and pemetrexed. A study on the latter combination therapy is currently being conducted (KEYNOTE-789, U. S. National Library of Medicine/ClinicalTrials.gov 2018). An assessment was conducted that used German-specific costs and care limiting the transferability to other countries.

## Conclusion

Targeted therapies are becoming increasingly important for the treatment of patients with NSCLC. Since treatment with palliative intent is the main focus at an advanced stage, the aim is to achieve both long PFS and OS with a minimum of side effects. In conclusion, the integration of LB as an add-on into the care pathway of advanced NSCLC has positive clinical effects in terms of PFS, OS and QALYs. Furthermore, its use is characterized by a moderate cost effectiveness, depending on the genetic alteration. For the total cohort, only small cost differences were observed. The beneficial potential is most significant for EGFR mutations. In general, the clinical benefit of LB information for subgroups may be substantial if diagnostic information cannot be obtained by other alternatives (example TB not feasible). Future research should focus on the further potential of LB to broaden the range of clinically relevant molecular information. This may include capturing molecular tumour heterogeneity or monitoring of minimal residual disease resulting from ongoing clonal evolution. Using LB in a close-meshed pattern may result in earlier clinical decision making. In addition, LB is subject to further technical and procedural development. Related improvements may add to its accuracy, precision, and ideally may drive down cost of its application. A consistent digital capturing of diagnostic information and resulting clinical outcome data is inevitable to understand clinical relevance and appropriate use patterns also from an economic perspective. Related savings can improve allocative efficiency and free resources for further innovation. As the translation of advances in the still devastating stage IV disease to non-metastatic NSCLC is moving into the focus of research, it is of pivotal importance to use innovative diagnostic tools for broad molecular profiling on a regular basis to fully exploit therapeutic potential in NSCLC patient care.

## Supplementary Information

Below is the link to the electronic supplementary material.

Supplementary file1 (DOCX 534 KB)

## Data Availability

The data sets analysed during the current study are available from the corresponding author on reasonable request.
