# Supplementary material for: Clinical benefit and cost-effectiveness analysis of liquid biopsy application in patients with advanced non-small cell lung cancer (NSCLC): a modelling approach
Source: J Cancer Res Clin Oncol. 2022 May 9;149(4):1495–511. doi: 10.1007/s00432-022-04034-w (PMC10020305; doi:10.1007/s00432-022-04034-w)
Supplement: Supplementary file 1 — Supplementary file1 (DOCX 534 KB) [file 432_2022_4034_MOESM1_ESM.docx]

**Supplement****ary Information**

**Clinical Benefit and Cost-Effectiveness Analysis of Liquid Biopsy Application in Patients with Advanced Non-Small Cell Lung Cancer (NSCLC) – A Modelling Approach**

Journal of Cancer Research and Clinical Oncology

**Table of contents**

[**Figure S.1** Treatment regimens used for modelling 1](#_Toc95218783)

[**Figure S.2** Flow diagram of literature research 2](#_Toc95218784)

[**Table S.1** Search strings for systematic literature research 3](#_Toc95218790)

[**Table S.2** Progression-free survival and overall survival for modelled first- and second-line treatments 4](#_Toc95218791)

[**Table S.3** Utilities depending on drug side effects 6](#_Toc95218792)

[**Table S.4** Utilities used for respective treatment lines and best supportive care 6](#_Toc95218793)

[**Table S.5** Utilities used for treatment line and respective medication 7](#_Toc95218794)

[**Table S.6** Treatment modalities and cost per year 8](#_Toc95218795)

[**Table S.7** Parameters for modelling 12](#_Toc95218796)

[Background information CRISP 15](#_Toc95218540)

[References 21](#_Toc95218541)

*
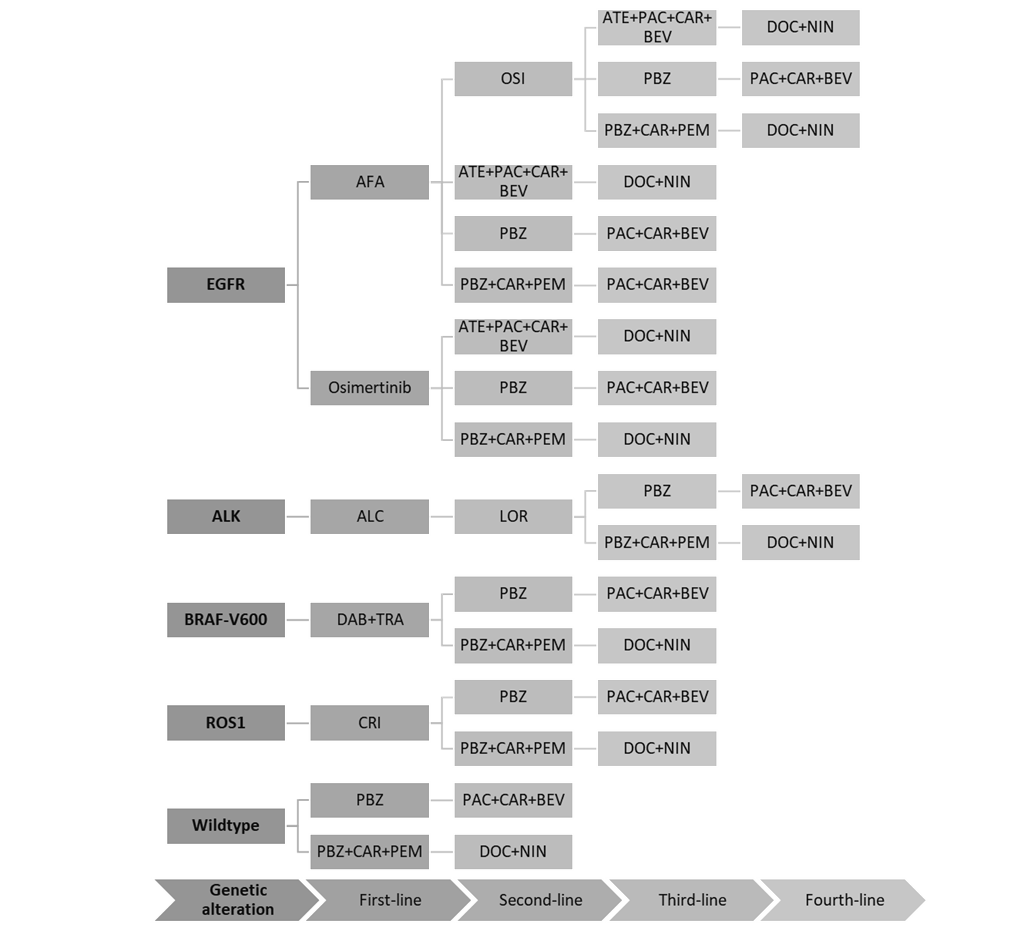
*

**Figure S.1** Treatment regimens used for modelling

Abbreviations: AFA = afatinib; ALC = alectinib; ATE = atezolizumab; BEV = bevacizumab; CAR = carboplatin; CRI = crizotinib; DAB = dabrafenib; DOC = docetaxel; LOR = lorlatinib; NIN = nintedanib; OSI = osimertinib; PAC = paclitaxel; PBZ = pembrolizumab; PEM = pemetrexed; TRA = trametinib

**Systematic literature research**

The databases PubMed (Medline) and Cochrane Library were used for articles published between 2010 and 2020. The terms NSCLC, the disease-specific genetic alteration (EGFR, ALK, BRAF-V600, ROS1) and the relevant treatment regimens (for non targeted therapies) defined the search string. The search was limited to peer reviewed articles (clinical trials) written in English and German. In addition, only studies with an available abstract and full text were included. The search strings and filters are given in **Table S.1**. References of relevant publications were assessed in detail to identify further studies. In addition, the inclusion criteria were met by studies reporting the PFS and OS of a target population diagnosed with metastatic NSCLC. Randomized controlled trials of phase III were included wherever possible. If no studies with a corresponding evidence level were available for the respective treatment regimens, phase I and II studies were used. The respective flow-diagram is depicted in **Fig. S.2.**


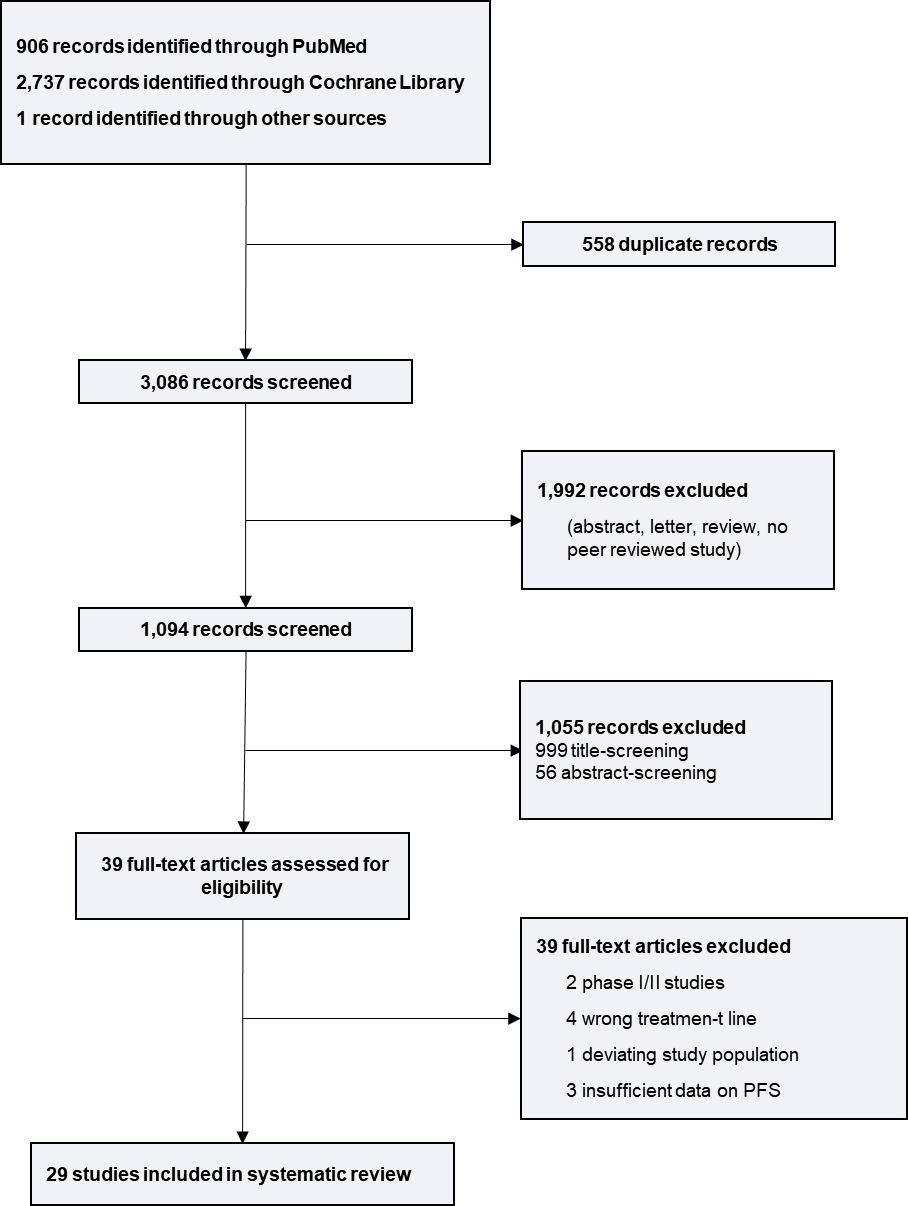


**Figure S.2** Flow diagram of literature research

**Table S.1** Search strings for systematic literature research

| Medline | Search | Hits |
| --- | --- | --- |
| #1 | Lung carcinoma, non small cell[MeSH Terms] | 55.602 |
| #2 | NSCLC[Title/Abstract] | 44.873 |
| #3 | Non small cell lung cancer[Title/Abstract] | 56.233 |
| #4 | (((#1) OR #2) OR #3) | 74.453 |
| #5 | ALK[Title/Abstract] | 9.607 |
| #6 | EGFR[Title/Abstract] | 60.243 |
| #7 | ROS1[Title/Abstract] | 1.334 |
| #8 | BRAF[Title/Abstract] | 15.370 |
| #9 | pembrolizumab[Title/Abstract] | 3.448 |
| #10 | docetaxel[Title/Abstract] | 14.908 |
| #11 | nintedanib[Title/Abstract] | 787 |
| #12 | atezolizumab[Title/Abstract] | 890 |
| #13 | bevacizumab[Title/Abstract] | 15.859 |
| #14 | paclitaxel[Title/Abstract] | 29.126 |
| #15 | carboplatin[Title/Abstract] | 14.651 |
| #16 | ((#10) AND #11) | 63 |
| #17 | ((#12 AND #13) AND #14 AND #15) | 16 |
| #18 | (((#13) AND #14) AND #15) | 475 |
| #19 | ((((((((#5) OR #6) OR #7) OR #8) OR #9) OR #16) OR #17) OR #18) | 84.534 |
|  | (((#4) AND #19) | 13.419 |
| #20 | (((#4) AND #19); Filters: (2010:2020[pdat]) | 11.858 |
| #21 | (((#4) AND #19); Filters: (2010:2020[pdat]); Clinical trial | 927 |
| #22 | (((#4) AND #19) AND: (2010:2020[pdat]); Filters Clinical trial; Humans | 925 |
|  | (((#4) AND #19) AND: (2010:2020[pdat]); Filters Clinical trial; Humans; English | 904 |
|  | (((#4) AND #19) AND: (2010:2020[pdat]); Filters Clinical trial; Humans; English; German | 906 |
| Cochrane library | Search | Hits |
| #1 | Non small cell lung cancer | 14.441 |
| #2 | NSCLC | 9.828 |
| #3 | ((#1) OR #2) | 15.175 |
| #4 | ALK | 1.417 |
| #5 | EGFR | 9.939 |
| #6 | ROS1 | 129 |
| #7 | BRAF | 1.345 |
| #8 | pembrolizumab | 1.851 |
| #9 | docetaxel | 7.538 |
| #10 | nintedanib | 582 |
| #11 | atezolizumab | 920 |
| #12 | bevacizumab | 6.578 |
| #13 | paclitaxel | 10.724 |
| #14 | carboplatin | 7.481 |
| #15 | ((#10) AND #11) | 42 |
| #16 | (((#11) AND #12) AND #13) AND #14) | 67 |
| #17 | (((#12) AND #13) AND #14) | 619 |
| #18 | ((((((((#4) OR #5) OR #6) OR #7) OR #8) OR #15) OR #16) OR #17) | 14.070 |
| #19 | ((#3 AND #18) | 3.464 |
| #20 | ((#3 AND #18) AND: (2010:2020[pdat]) | 2.971 |
| #21 | ((#3 AND #18) AND: (2010:2020[pdat]); Filters Clinical trial | 2.926 |
| #22 | ((#3) AND #18) AND: (2010:2020[pdat]); Filters Clinical trial; custom year range 2010 to 2020 | 2.737 |

*Literature research 02.06.2020; Filters: last 10 years, Abstract, Full-Text, Clinical Trial*

**Table S.2** Progression-free survival and overall survival for modelled first- and second-line treatments

| **Alteration** | **Line** | **Medication** | **Number of patients^a^** | **Median PFS in months**  **Range (95 % CI)** | **Median OS in months**  **Range (95 % CI)** | **Study Name** |
| --- | --- | --- | --- | --- | --- | --- |
| **First line** |  |  |  |  |  |  |
| EGFR | 1st-line | afatinib | 230 | 11.1  (9.6 to 13.7) | 28.2  (24.6-33.6)^b^ | LUX-Lung 3^1^ |
| EGFR | 1st-line | afatinib | 242 | 11.0  (9.7 to 13.7) | 23.1  (20.4-27.3)^b^ | LUX-Lung 6^2^ |
| EGFR | 1st-line | osimertinib | 279 | 18.9  (15.2 to 21.4) | 38.6  (34.5-41.8) | FLAURA^3,4^ |
| ALK | 1st-line | crizotinib | 172 | 10.9  (8.3 to 13.9) | NE  (45.8-NE) | PROFILE 1014^5-7^ |
| ALK | 1st-line | crizotinib | 151 | 10.9  (9.1 to 13.0) | NE | ALEX^8-10^ |
| ALK | 1st-line | crizotinib | 127 | 11.1  (8.3 to 12.6) | 28.5  (26.4-NE) | PROFILE 1029^11^ |
| ALK | 1st-line | crizotinib | 62 | 11.1  (9.1 to 13.0) | NE | ALESIA^12^ |
| ALK | 1st-line | alectinib | 152 | 34.8  (17.7 to NE) | NE | ALEX^8-10^ |
| ROS1 | 1st-line | crizotinib | 53 | 19.3  (15.2-39.1) | 51.4  (29.3-NE) | PROFILE 1001^13,14^ |
| BRAF-V600 | 1st-line | dabrafenib+ trametinib | 36 | 10.9  (7.0 to 16.6) | 24.6  (12.3-NE) | -^15^ |
| PDL1 ≥ 50 % | 1st-line | pembrolizumab | 154 | 7.7  (6.1-10.2) | 26.3  (18.3-40.4) | KEYNOTE-024^16,17^ |
| PDL1 ≥ 50 % | 1st-line | pembrolizumab | 637 | 7.1  (5.9-9.0) | 20.0  (15.4-24.9) | KEYNOTE-042^18^ |
| PDL1 1-49 % | 1st-line | pembrolizumab+ pemetrexed+ carboplatin | 128 | 9.2  (7.8 to 13.1) | 21.8  (17.7-25.9) | KEYNOTE-189^19,20^ |
| regardless PDL1 | 1st-line | pembrolizumab+ pemetrexed+ carboplatin | 127 | 6.2  (4.9 to 8.1) | 17.2  (13.8-22.8) | KEYNOTE-189^19,20^ |
| **Second line** |  |  |  |  |  |  |
| EGFR (T790M) | 2nd-line | osimertinib | 279 | 10.1  (8.3 to 12.3) | 26.8  (23.5-31.5)^b^ | AURA3^21^ |
| ALK | 2nd-line | alectinib | 79 | 9.6  (6.9 to 12.2) | 27.8  (18.2-NE) | ALUR^22^ |
| ALK | 2nd-line | lorlatinib | 28 | 5.5  (2.7-9.0) | NE  (14.4-NE) | -^23^ |
| **Alteration** | **Line** | **Medication** |  | **Median PFS in months**  **Range (95 % CI)** | **Median OS in months**  **Range (95 % CI)** | **Study Name** |
| subgroup EGFR | 2nd-line | atezolizumab+ paclitaxel+ carboplatin+ bevacizumab | 34 | 9.7  (NE) | NE  (17.0-NE) | IMpower150^24,25^ |
| regardless PDL1 | 2nd-line | docetaxel+ nintedanib | 655 | 4.2  (2.1-7.1) | 12.6  (5.5-24.2) | LUME-Lung 1^26,27^ |
| PDL1 ≥ 50 % | 2nd-line | pembrolizumab | 290 | 5.2  (4.0-6.5) | 14.9  (10.4-NE) | KEYNOTE-010^28,29^ |
| subgroup EGFR | 2nd-line | paclitaxel+ carboplatin+ bevacizumab | 45 | 6.1  (NE) | 18.7  (13.4-NE) | IMpower150^24,25^ |

^a^*The number of patients received the medication listed in column three*

^b^*Published studies were matched with data from clinicaltrials.gov. Data marked with* ^b^ *represent updated values identified in the U. S. National Library.*

*The study by Reck et al. (2021) KEYNOTE-024 was published outside the time frame specified for the review. However, as the data have an impact on the results of the cost-effectiveness analysis, the updated values of the 2021 publication were used for the median PFS.*

**Table S.3** Probabilities of drug side effects

| **Drug** | **Study name** | **Diar-rhea** | **Fati-gue** | **Febrile neutro-penia** | **Hair loss** | **Nausea/Vomi-ting** | **Neutro-penia** | **Rash** | **Bleed-ing** | **Hyper-tension** | **ORR** |
| --- | --- | --- | --- | --- | --- | --- | --- | --- | --- | --- | --- |
| AFA | (LUX-Lung 6) | 0.054 | 0.004 | 0 | 0 | 0.008 | 0.004 | 0.146 | 0 | 0 | 0.678 |
| OSI  (2nd-line) | (AURA3) | 0.01 | 0.01 | 0 | 0 | 0.01 | 0 | 0.01 | 0 | 0 | 0.71 |
| PBZ  (1st line) | KEYNOTE-024 | 0.039 | 0.019 | 0 | 0 | 0 | 0 | 0.013 | 0 | 0 | 0.448 |
| PBZ  (2nd-line) | KEYNOTE-010 | 0.003 | 0.015 | 0 | 0 | 0.004 | 0 | 0.003 | 0 | 0 | 0.29 |
| PBZ+CAR+PEM | KEYNOTE-189 | 0.052 | 0.069 | 0 | 0 | 0.04 | 0.16 | 0.02 | 0 | 0 | 0.323 |
| ABCP | IMpower150 | 0.028 | 0.033 | 0.092 | 0 | 0.038 | 0.137 | 0.013 | 0 | 0.064 | 0.706 |
| BCP | IMpower150 | 0.005 | 0.025 | 0.058 | 0 | 0.038 | 0.112 | 0 | 0 | 0.063 | 0.419 |
| DOC+NIN | LUME-Lung 1 | 0.067 | 0.057 | 0.07 | 0.002 | 0.008 | 0.121 | 0 | 0.013 | 0 | 0.047 |
| ALC | ALEX | 0 | 0 | 0 | 0 | 0.01 | 0 | 0 | 0 | 0 | 0.829 |
| LOR | Solomon et al. | 0.004 | 0.004 | 0 | 0 | 0.004 | 0 | 0.004 | 0 | 0.01 | 0.321 |
| CRI  (ROS1) | PROFILE1001 | 0 | 0 | 0 | 0 | 0.06 | 0.09 | 0 | 0 | 0 | 0.72 |
| DAB+TRA | Planchard et al. | 0.03 | 0 | 0 | 0 | 0.08 | 0.03 | 0.06 | 0.03 | 0 | 0.64 |
| OSI  (1st-line) | FLAURA | 0.03 | 0.06 | 0 | 0.01 | 0 | 0 | 0 | 0 | 0 | 0.8 |

**Table S.4** Utilities used for respective treatment lines and best supportive care

|  | **NSCLC intercept^30^** | **First-line^31^** | **Second-line^30^** | **Third- and fourth-line^32^** | **Best Supportive Care^32^** |
| --- | --- | --- | --- | --- | --- |
| Stable Disease (LQ_S_) | 0.653 | 0.814 | 0.653 | 0.620 | 0.46 |
| Response (LQ_R_) |  | 0.854 | 0.673 | 0.620 | 0.46 |

**Table S.5** Utilities used for treatment line and respective medication

| **First-line** | **Stable/Responding** | **Progression** |
| --- | --- | --- |
| AFA | 0.797 | 0.617 |
| ALC | 0.845 | 0.665 |
| CRI | 0.785 | 0.605 |
| DAB+TRA | 0.780 | 0.600 |
| OSI | 0.809 | 0.629 |
| PBZ | 0.808 | 0.629 |
| PBZ+CAR+PEM | 0.690 | 0.511 |
| **Second-line** | **Stable/Responding** | **Progression** |
| ABCP | 0.511 | 0.331 |
| BCP | 0.550 | 0.370 |
| DOC+NIN | 0.503 | 0.323 |
| LOR | 0.6544 | 0.475 |
| OSI | 0.655 | 0.476 |
| PBZ | 0.650 | 0.470 |
| PBZ+CAR+PEM | 0.523 | 0.343 |
| **Third- and fourth-line** | **Stable/Responding** | **Progression** |
| PBZ | 0.611 | 0.431 |
| PBZ+CAR+PEM | 0.484 | 0.304 |
| DOC+NIN | 0.469 | 0.289 |
| ABCP | 0.464 | 0.284 |
| BCP | 0.509 | 0.329 |

Abbreviations: AFA = afatinib; ALC = alectinib; ATE = atezolizumab; BEV = bevacizumab; CAR = carboplatin; CRI = crizotinib; DAB = dabrafenib; DOC = docetaxel; LOR = lorlatinib; NIN = nintedanib; OSI = osimertinib; PAC = paclitaxel; PBZ = pembrolizumab; PEM = pemetrexed; TRA = trametinib

The calculation was carried out using the data from Table S.2 and Table S.3 based on the following formula:

Life quality (LQ) =

LQ_S_ +((LQ_R_ – LQ_S_) x pORR + pDiarrhoa x (-0.34^31^) + pFatigue x (-0.44^31^) + pFebrileNeutropenia x (-0.59^31^) + pHairLoss x (-0.09^31^) + pNausea/Vomiting x (-0.26^31^) + pNeutropenia x (-0.47^31^) + pRash x (-0.14^31^) + pBleeding x (-0.21^31^) + pHypertension x (-0.03^31^) + pPneumothorax x (-0.04^33^) + pProgress x (-0.1798^30^))

**Table S.6** Treatment modalities and cost per year

| **Medication** | **Treatment mode** | **Dose per day** | **Size of package** | **Consumption per year (packages or vials)** | **Cost per vial/pack-age (in €)** | **Costs with discounts**  **(in €)** | **Additional cost (in €)^a^** | **Treatment cost per year (in €)** |
| --- | --- | --- | --- | --- | --- | --- | --- | --- |
| alectinib | continuously (365 days) | 1200 mg: 2x 600 mg | ALCENSA® 224 pills à 150 mg | 13.04 | 5,976.63 | 5,636.81 | 0.00 | 73,479.84 |
| afatinib | continuously (365 days) | 1x 40 mg | GIOTRIF® 28 pills à 40 mg | 13.04 | 2,514.99 | 2,372.87 | 0.00 | 30,932.06 |
| osimertinib | continuously (365 days) | 1x 80 mg | TAGRISSO® 30 pills à 80 mg | 12.17 | 6,155.92 | 5,805.86 | 0.00 | 70,637.96 |
| crizotinib | continuously (365 days) | 500 mg: 2x 250 mg | XALKORI® 60 pills à 250 mg | 12.17 | 5,425.95 | 5,424.18 |  | 65,994.19 |
| lorlatinib | continuously (365 days) | 1x 100 mg | LORVIQUA® 30 pills à 100 mg | 12.17 | 7,815.46 | 7,370.62 |  | 89,675.88 |
| trametinib | continuously (365 days) | 1x 2 mg | MEKINIST® 30 pills à 2 mg | 12.17 | 4,367.34 | 4,365.57 |  | 53,114.44 |
| dabrafenib | continuously (365 days) | 2x 150 mg | TAFINLAR® 120 pills à 75 mg | 12.17 | 5,831.71 | 5,829.94 |  | 70,930.94 |
| pembrolizumab |  |  |  |  |  |  |  | ***98,746.20*** |
| pembrolizumab | 1 time per 21 day cycle (17 cycles) | 200 mg; 2 vials of 100 mg | KEYTRUDA® 25mg/ml 4ml | 34.00 | 3,037.06 | 2,865.12 | 1,207.00^b^ | 98,621.08 |
| Infusion |  |  |  |  |  |  | 125.12 | 125.12 |
| ***PBZ+CAR+PEM*** |  |  |  |  |  |  |  | ***171,006.11*** |
| pembrolizumab | 1 time per 21 day cycle (17 cycles) | 200 mg; 2 vials of 100 mg | KEYTRUDA® 25mg/ml 4ml | 34.00 | 3,037.06 | 2,865.12 | 1,207.00 ^b^ | 98,621.08 |
| **Medication** | **Treatment mode** | **Dose per day** | **Size of package** | **Consumption per year (packages or vials)** | **Cost per vial/package (in €)** | **Costs with discounts**  **(in €)** | **Additional cost (in €)^a^** | **Treatment cost per year (in €)** |
| carboplatin | 1 time every 21 days (6 cycles) | 500 mg/m^2^ ≙ 950 mg: |  |  |  |  | 486.00 ^c^ | 486.00 |
|  |  | 1x 450 mg | BENDALIS®1 vial à 450 mg | 6.00 | 227.97 | 215.91 | 0.00 | 1,295.46 |
|  |  | 1x 600 mg | BENDALIS®1 vial à 600 mg | 6.00 | 300.57 | 285.06 | 0.00 | 1,710.36 |
| pemetrexed | 1 time per 21 day cycle (17 cycles) | 500 mg/m^2^ ≙ 950 mg: 2x 500 mg | ALIMTA® 1 vial à 500 mg | 34.00 | 2,533.30 | 1,972.89 | 1,506.74^d^ | 68,585.00 |
| infusion (combi-nation therapy) |  |  |  |  |  |  | 308.21 | 308.21 |
| Infusion (maintenance therapy) |  |  |  |  |  |  | 125.12 | 125.12 |
| ***ATE+BEV+ CAR+PAC*** |  |  |  |  |  |  |  | ***170,586.43*** |
| bevacizumab | 1 time every 21 days (17 cycles) | 15 mg/kg ≙1155 mg: 3x 400 mg | AVASTIN® 1 vial à 400mg | 51.00 | 1,689.86 | 1,594.86 | 1,207.00 ^b^ | 82,544.86 |
| atezolizumab | 1 time every 21 days (17 cycles) | 1200 mg | TECENTRIQ® 1vial à 1200 mg | 17.00 | 4,692.05 | 4,425.59 | 1,207.00 ^b^ | 76,442.03 |
| carboplatin | 1 time every 21 days (6 cycles) | 500 mg/m^2^ ≙ 950 mg: |  |  |  |  | 486.00 ^c^ | 486.00 |
|  |  | 1x 450 mg | BENDALIS® 1 vial à 450 mg | 6.00 | 227.97 | 215.91 | 0.00 | 1,295.46 |
|  |  | 1x 600 mg | BENDALIS®1 vial à 600 mg | 6.00 | 300.57 | 285.06 | 0.00 | 1,710.36 |
| **Medication** | **Treatment mode** | **Dose per day** | **Size of package** | **Consumption per year (packages or vials)** | **Cost per vial/package (in €)** | **Costs with discounts**  **(in €)** | **Additional cost (in €)^a^** | **Treatment cost per year (in €)** |
| paclitaxel | 1 time every 21 days (6 cycles) | 175 mg/m^2^ ≙ 332,5 mg: |  |  |  |  | 547.97 ^e^ | 547.97 |
|  |  | 2x 30 mg | PACLITAXEL Kabi® 1 vial à 30 mg | 12.00 | 113.70 | 107.06 |  | 1,284.72 |
|  |  | 1x 300 mg | CELLTAXEL® 1 vial à 300 mg | 6.00 | 1,045.32 | 994.47 |  | 5,966.82 |
| infusion |  |  |  |  |  |  | 308.21 | 308.21 |
| ***DOC+NIN*** |  |  |  |  |  |  |  | ***54,882.79 €*** |
| docetaxel | 1 time per 21 day cycle (17 cycles) | 75 mg/m^2^ ≙ 142.5 mg | AXIOS® 1 vial à 160 mg | 17.00 | 1,397.36 | 1,220.15 | 1,496.11 ^f^ | 22,238.66 |
| infusion/ clinical supervision |  |  |  |  |  |  | 827.39 | 827.39 |
| nintedanib | 20 times per cycle (17 cycles) | 400 mg: 2x 200 mg | VARGATEF® 60 pills à 100 mg | 22.67 | 2,761.03 | 1,403.68 |  | 31,816.75 |
| ***PAC+CAR+BEV*** |  |  |  |  |  |  |  | ***94,144.40*** |
| carboplatin | 1 time every 21 days (6 cycles) | 500 mg/m^2^ ≙ 950 mg: |  |  |  |  | 486.00 ^c^ | 486.00 |
|  |  | 1x 450 mg | BENDALIS®1 vial à 450 mg | 6.00 | 227.97 | 215.91 | 0.00 | 1,295.46 |
|  |  | 1x 600 mg | BENDALIS®1 vial à 600 mg | 6.00 | 300.57 | 285.06 | 0.00 | 1,710.36 |
| **Medication** | **Treatment mode** | **Dose per day** | **Size of package** | **Consumption per year (packages or vials)** | **Cost per vial/package (in €)** | **Costs with discounts**  **(in €)** | **Additional cost (in €)^a^** | **Treatment cost per year (in €)** |
| paclitaxel | 1 time every 21 days (6 cycles) | 75 mg/m^2^ ≙ 332,5 mg: |  |  |  |  | 547.97 ^e^ | 547.97 |
|  |  | 2x 30 mg | PACLITAXEL Kabi® 1 vial à 30 mg | 12.00 | 113.70 | 107.06 |  | 1,284.72 |
|  |  | 1x 300 mg | CELLTAXEL® 1 vial à 300 mg | 6.00 | 1,045.32 | 994.47 |  | 5,966.82 |
| bevacizumab | 1 time every 21 days (17 cycles) | 15 mg/kg ≙1155 mg: 3x 400 mg | AVASTIN® 1 vial à 400mg | 51.00 | 1,689.86 | 1,594.86 | 1,207.00 ^b^ | 82,544.86 |
| infusion |  |  |  |  |  |  | 308.21 | 308.21 |

*Drug prices are in accordance with the German Lauer-Taxe (as of 17.06.2020). Reimbursement rates for statutory health insurance were extracted from the respective* *reimbursement catalogue: “Einheitlicher Bewertungsmaßstab (EBM)”; for dosage calculation we assumed a body surface area (BSA) of 1.90 m^2^. The calculated dosage for patients is based on the average height (1.72 m) and weight (77 kg) of an adult in Germany.^34^*

*^a^ solution for infusion intravenous use and accompanying therapies*

^b^ *solution for infusion intravenous use (€ 71 per cycle)*

^c^ *solution for infusion intravenous use (€81 per cycle)*

^d^ *solution for infusion intravenous use (€81 per cycle) + accompanying therapies (dexamethason + folverlan + vitamin B12)*

^e^ *solution for infusion intravenous use (€81 per cycle) + accompanying therapies (diphenhydramin + ranitidin)*

^f^ *solution for infusion intravenous use (€81 per cycle) + accompanying therapies (dexamethason + folverlan)*

**Table S.7** Parameters for modelling

| **Parameter** | **Value** | **Source** |
| --- | --- | --- |
| **Cost** |  |  |
| per case payment (ambulatory visit) | €23.18 | GOP 13642^35^ |
| oncological charge | €20.99 | GOP 13675^35^ |
| cost large panel liquid biopsy | €3,332.00 | FoundationOne® Liquid (Foundation Medicine) |
| cost large panel tissue biopsy | €3,498.62 | GOP 19401-19404, GOP 19454^35^ |
| cost mutation analysis in up to 20 kilobases of coding sequence tissue biopsy | €278.63 | GOP 19401-19404, GOP 19453 |
| Detection or exclusion of all known EGFR-activating mutations in exons 18 to 21 by liquid biopsy | €535.95 | GOP 19401-19403, GOP 19461 |
| cost tissue biopsy |  |  |
| inpatient (80 %)^a^ | €2.700.00 | Inpatient: G-DRG E71C^b^ |
| outpatient (20 %)^a^ | €280.00 | GOP 13650, 13651, 13642, 13662, 13663, 32247^c^ |
| **Choice of treatment line** |  |  |
| proportion of first-line afatinib (EGFR) | 0.21 | ^36^ |
| proportion of first-line osimertinib (EGFR) | 0.79 | ^36^ |
| proportion of second-line treatment (ALK) | 0.39-0.82 | ^36^ |
| proportion of second line treatment (BRAF-V600) | 0.18-1 | ^36^ |
| proportion of second-line treatment (EGFR) | 0.44-0.8 | ^36^ |
| proportion of second-line treatment (no oncogenic driver) | 0.47-0.68 | ^36^ |
| proportion of second-line treatment (PD-L1 ≥ 50 %) | 0.31-0.7 | ^36^ |
| proportion of second-line treatment (PD-L1 1 %-49 %) | 0.47-0.68 | ^36^ |
| proportion second-line treatment (ROS1) | 0.67-0.89 | ^36^ |
| proportion second-line atezolizumab combination | 0.2 | ^a^ |
| proportion ALK translocation | 0.02 | ^37^ |
| proportion of BRAF-V600 mutation | 0.02 | ^37^ |
| proportion of EGFR mutation | 0.13 | ^37^ |
| proportion of ROS1 rearrangement | 0.01 | ^37^ |
| proportion positive PD-L1 expression | 0.69 | ^36^ |
| proportion PD-L1 expression ≥ 50 % | 0.37 | ^36^ |
| proportion of PD-L1 expression 1-49 % | 0.63 | ^36^ |
| proportion resistance T790M | 0.55 | ^38-42^ |
| **Parameter** | **Value** | **Source** |
| **Feasibility of tissue and molecular analysis** |  |  |
| probability of inadequate tissue for molecular testing after tissue biopsy | 0.2 | ^43-47^ |
| probability repeated tissue biopsy is not feasible | 0.195 | ^47^ |
| failure rate in molecular pathological examination based on plasma | 0.09 | ^48^ |
| **Sensitivity** |  |  |
| Sen_ALK_LB | 1  (95 % CI 0.561-1) | ^49^ |
| Sen_ALK_TB | 0.99 | ^50^ |
| Sen_BRAF_LB | 0.957  (95 % CI 0.949-0.964) | ^49^ |
| Sen_BRAF_TB | 0.99 | ^50^ |
| Sen_EGFR_LB | 0.92  (95 % CI 0.814-0.964) | ^49^ |
| Sen_EGFR_TB | 0.97 | ^50^ |
| Sen_ROS1_LB | 1  (95 % CI 0.561-1) | ^49^ |
| Sen_ROS1_TB | 0.99 | ^50^ |
| Sen_T790M_LB | 0.957  (95 % CI 0.949-0.964) | ^49^ |
| Sen_T790M_TB | 0.99 | ^50^ |
| **Median progression-free survival** |  |  |
| AFA (first-line) | 11.05 | Pooled effect: LUX-Lung 3^1,2^, LUX-Lung 6^2^ |
| ALC (first-line) | 34.8 | ALEX^8-10^ |
| ALC (second-line) | 9.6 | ALUR^22^ |
| ATZ+CAR+PEM+BEV (second line) | 9.7 | IMpower150^24,25^ |
| CAR+PAC+BEV (second line) | 6.1 | IMpower150^24,25^ |
| CRI (ALK) (first-line) | 11.0 | Pooled effect: PROFILE 1029^11^, ALEX^8-10^, PROFILE 1014^5-7^, ALESIA^12^ |
| CRI (ROS1) | 19.3 | PROFILE 1001^13,14^ |
| DAB+TRA (first-line) | 10.9 | -^15^ |
| DOC+NIN (second-line) | 4.2 | LUME-Lung 1^26,27^ |
| LOR (second-line) | 8.2 | -^23^ |
| OSI (first-line) | 18.9 | FLAURA^3,4^ |
| OSI (second-line) | 10.1 | AURA 3^21^ |
| PBZ (PDL1 ≥ 50 % first-line) | 7.3 | Pooled effect: KEYNOTE-024^16,17^, KEYNOTE-042^18^ |
| **Parameter** | **Value** | **Source** |
| PBZ (PDL1 ≥ 50 % second-line) | 5.2 | KEYNOTE-010^28,29^ |
| PBZ+PEM+CAR (PDL1 1-49 % first line) | 9.2 | KEYNOTE-189^19,20^ |
| PBZ+PEM+CAR (regardless of PDL1 first line) | 6.2 | KEYNOTE-189^19,20^ |

*^a^Assumption of experts*

*^b^Includes OPS codes for endoscopic biopsy of respiratory organs (OPS 1-430.1) and diagnostic tracheobronchoscopy with flexible instrument (OPS 1-620.00); Assumption: inpatient stay of two days and one ambulatory visit*

*^c^ In accordance with the German fee schedule (EBM Codes)*

*Abbreviations:* *AFA = afatinib; ALC = alectinib; ATE = atezolizumab; BEV = bevacizumab; CAR = carboplatin; CRI = crizotinib; DAB = dabrafenib; DOC = docetaxel; GOP = Gebührenordnungsposition (codes of the German reimbursement catalogue “Einheitlicher Bewertungsmaßstab (EBM)”);* *LB = liquid biopsy; LOR = lorlatinib; NIN = nintedanib; OSI = osimertinib; PAC = paclitaxel; PBZ = pembrolizumab; PEM = pemetrexed; TB = tissue biopsy; TRA = trametinib*

# **Background information CRISP**

Extracts from the German CRISP-Project - AIO-TRK-0315 were used for modelling purposes. Data on:

- Proportion second-line therapies (data of the year 2018 were used for modelling)
- Frequencies of top used regimes by the underlying druggable alteration (data of the year 2019 were used for modelling)
- Proportion of PD-L1 expression

1. **Objectives and Results**

***Objectives of this analysis:***
The overall aim of the prospective cohort analysis is to assess proportion and type of 2nd-line treatment of patients with locally advanced or metastatic non-small cell lung cancer (stage IIIB and IV NSCLC) and with varying underlying druggable gene alterations.
Aims

- To calculate the proportion of second-line treatments
- To describe the most frequently used treatment regimens by the underlying druggable gene alteration
- To describe the proportions of patients with PD-L1 expression ≥50 % and between 1 % and 49 %

Analysis Populations
The analysis will be performed in the following patient (sub-)populations (both, non-squamous and squamous NSCLC):

- Full Analysis Set (FAS): All eligible patients meeting the study inclusion criteria (CRISP NSCLC later stage cohort).
- Druggable EGFR Mutation Analysis Set (EGFR): A sub-set of the study cohort, patients with druggable EGFR mutations including all patients with a documented test result defined as deletion 19, L858R, T790M, and/or group I (point mutations and duplications, or both, in exons 18-21).
- Druggable ALK Mutation Analysis Set (ALK): A sub-set of the study cohort, patients with druggable ALK mutations defined as translocation, or with FISH positive or IHC positive (with IHC Detail + to +++, or unknown or not specified).
- Druggable ROS1 Mutation Analysis Set (ROS1): A sub-set of the study cohort, patients with druggable ROS1 mutations defined as translocation, or as FISH positive.
- Druggable BRAF Mutation Analysis Set (BRAF): A sub-set of the study cohort, patients with a druggable BRAF mutation documented as „V600” mutation.
- PD-L1 positive ≥50% Analysis Set (PD-L1>50%): A sub-set of the study cohort, patients with confirmed positive PD-L1 expression test result with equal or more than 50% of PD-L1 positive tumour cells.
- No clinically relevant driver Analysis Set (no DrLT): A sub-set of the study cohort, patients with no drug licensed target detected or patients not tested.

1. **Results**

***Proportion of second-line therapies***

However, it has to be considered that the proportion of patients who started a 2nd-line describes the minimum frequency of patients receiving 2nd-line therapy. The proportion of patients who died after/during 1st-line therapy defines the limit for the maximum frequency of patients receiving 2nd-line therapy. For patients whose line of therapy was still ongoing or whose new line of therapy had not yet started, there is still the possibility to receive or not receive 2nd-line therapy. Thus, the maximal proportion of patients receiving a 2nd-line is given by the proportion of patients with confirmed 2nd-line plus the proportion of patients who of could potentially receive a 2nd treatment.

***Most frequently used treatment regimes***

The two most frequent targeted treatment regimens for the different analysed subgroups are used for modelling. The top two regimens might vary between the most recent and previous years, e.g. because of approval of new drugs. However, it has to kept in mind that case numbers for the ROS1 and BRAF druggable cohorts are very small (<15 per year) hindering reliable interpretation of changes over time.

***Proportion of patients with PD-L1 expression in the CRISP NSCLC cohort***

The proportion of patients with a positive test result for PD-L1 expression and details on test results documented at the time of database cut are used. For this analysis, data from December 2015 to June 2020 are included.

***Limitations***
This analysis of data from the CRISP later stage NSCLC cohort presents the treatment reality of patients enrolled into the CRISP project from 2017 to June 30th, 2020. Therefore, the follow-up time of about one third of the patients was at this time point limited to less than one year. Not all first-line treatments that had been started within this time had been finished at database cut of this analysis. Therefore, the proportions of 2nd-line treatments for 2019 and 2020 are currently underrepresented and will rise with longer follow-up. Thus, the currently documented minimum and maximum number of patients who received and could receive second-line treatments should be considered the best estimate of treatment reality.

The most frequently applied regimens can change over time, e.g. with the approval of new treatments. However, small number of patients per most recent time period can hinder reliable interpretation because seemingly large changes in percentages can be due to a few patients. Thus both, the number of patients per time period and potential changes over time should be considered when interpreting the data. Most importantly, case numbers for the BRAF and ROS1 druggable alteration groups are very small and that has to be considered when interpreting the data.

Since CRISP is an ongoing project and outcome data for specific subgroups are still based on a small number of patients, this should be strongly considered when interpreting or comparing the data with other sources. Furthermore, when comparing outcome data from CRISP with published data from clinical trials it has to be considered that patients and assessment methodologies and frequencies differ between clinical trials and routine care.

CRISP presents the treatment reality of systemically treated patients with locally advanced or metastatic nonsmall cell lung cancer (NSCLC) in Germany. All treatments are allowed, and no restrictions are imposed. Treatment decision making is with the treating physician and can depend on numerous factors. Therefore, it cannot be assumed that the patient population is homogeneous for different therapies.

1. **Design and Methods**

Data source: CRISP

The CRISP registry is an open, non-interventional, prospective, multi-centre clinical research platform on (non-) small cell lung cancer (NSCLC and SCLC). Patients with locally advanced or metastatic NSCLC at the start of palliative 1st-line systemic therapy were recruited into the later stage NSCLC cohort by up to 170 study sites
(certified lung cancer centres, comprehensive cancer centres, hospitals and office-based oncology practices) in Germany. The recruitment period for this cohort of the CRISP registry started in December 2015. Participating sites do not receive any specification on how to perform diagnostics or how to treat patients. The project’s physicians decide independently which treatments, dosage and concomitant medications, which methods for follow-up or which other therapeutic or diagnostic methods they apply. Thus, CRISP collects representative data on molecular testing, sequences of therapies and other treatment modalities, as well as the course of disease and gives insight into the treatment of unselected patients in routine practice. Data on patients’ demographics and clinical (tumour) characteristics as well as biomarker testing and previous (non-palliative) treatments were documented at inclusion. During the follow-up period (until death, or project end), data on additional molecular testing, all treatments, course of disease and outcome is updated at least every three months.

All data were transferred from medical charts into iOMEDICO's electronic documentation system. The electronic data capture system had implemented completeness and plausibility checks. In addition, key parameters were checked regularly by the data management and study sites contacted if necessary. The prospectively documented data from the CRISP registry is not linkable or linked to other registries or databases. CRISP has been reviewed by an independent ethics committee and is registered at Clinicaltrials.gov (NCT02622581).

***Study sites***

Participating study sites have an important role in assuring the quality of CRISP. In order to collect data representative for routine systemic treatment in Germany, a large number of certified lung cancer 8 centres, comprehensive cancer centres, hospitals and office-based oncology practices located all over Germany participate in CRISP. Study sites are encouraged to recruit patients consecutively.

***Methods***

For this study prospectively documented data from the CRISP registry were used. Database cut for this analysis was June 30th, 2020. All analyses were calculated using SAS software, Version 9.4 of the SAS System for Windows. Copyright © 2002- 2012 SAS Institute Inc. SAS and all other SAS Institute Inc. product or service names are registered trademarks or trademarks of SAS Institute Inc., Cary, NC, USA

***Patients***

CRISP later stage NSCLC cohort (FAS)

Inclusion Criteria: Patients who meet all of the following
criteria are eligible for the project:

- Confirmed non-small cell lung cancer (NSCLC)
- Stage IV or stage IIIB/C (UICC 8) if patient is ineligible for curative surgery and/or radiochemotherapy
- Informed consent no later than four weeks after start of systemic (palliative) 1^st^ line treatment
- Age ≥ 18 years
- Able to understand and willing to sign written Informed Consent and to complete patient-reported-outcome assessment instruments

Study population subgroups

The study population subgroups consist of CRISP patients from the FAS who meet one of the following additional criteria:

1. Inclusion criteria of CRISP later stage cohort (FAS) and
   1. patients with druggable EGFR mutations including all patients with a
      documented test result defined as deletion 19, L858R, T790M, and/or group I.
   2. patients with druggable ALK mutations defined as translocation, or with FISH positive or IHC positive (with IHC Detail + to +++, or unknown or not specified).
   3. patients with druggable ROS1 mutations defined as translocation, or as FISH positive
   4. patients with a druggable BRAF mutation documented as „V600” mutation
   5. patients with confirmed positive PD-L1 expression test result irrespective of documentation of percentage of PD-L1 positive tumour cells.
      1. patients with confirmed positive PD-L1 expression test result with more than 50% of percentage of PD-L1 positive tumour cells.
   6. patients with no drug licensed drug target detected (EGFR, ALK, ROS1,
      BRAF, PD-L1) or patients not tested.

References

1. Sequist LV, Yang JC-H, Yamamoto N, et al. Phase III study of afatinib or cisplatin plus pemetrexed in patients with metastatic lung adenocarcinoma with EGFR mutations. *J Clin Oncol*. 2013;31(27):3327-3334. doi:10.1200/JCO.2012.44.2806.

2. Wu Y-L, Zhou C, Hu C-P, et al. Afatinib versus cisplatin plus gemcitabine for first-line treatment of Asian patients with advanced non-small-cell lung cancer harbouring EGFR mutations (LUX-Lung 6): an open-label, randomised phase 3 trial. *Lancet Oncol*. 2014;15(2):213-222. doi:10.1016/S1470-2045(13)70604-1.

3. Soria J-C, Ohe Y, Vansteenkiste J, et al. Osimertinib in Untreated EGFR-Mutated Advanced Non-Small-Cell Lung Cancer. *N Engl J Med*. 2018;378(2):113-125. doi:10.1056/NEJMoa1713137.

4. Ramalingam SS, Vansteenkiste J, Planchard D, et al. Overall Survival with Osimertinib in Untreated, EGFR-Mutated Advanced NSCLC. *N Engl J Med*. 2020;382(1):41-50. doi:10.1056/NEJMoa1913662.

5. Solomon BJ, Mok T, Kim D-W, et al. First-line crizotinib versus chemotherapy in ALK-positive lung cancer. *N Engl J Med*. 2014;371(23):2167-2177. doi:10.1056/NEJMoa1408440.

6. Solomon BJ, Cappuzzo F, Felip E, et al. Intracranial Efficacy of Crizotinib Versus Chemotherapy in Patients With Advanced ALK-Positive Non-Small-Cell Lung Cancer: Results From PROFILE 1014. *J Clin Oncol*. 2016;34(24):2858-2865. doi:10.1200/JCO.2015.63.5888.

7. Solomon BJ, Kim D-W, Wu Y-L, et al. Final Overall Survival Analysis From a Study Comparing First-Line Crizotinib Versus Chemotherapy in ALK-Mutation-Positive Non-Small-Cell Lung Cancer. *J Clin Oncol*. 2018;36(22):2251-2258. doi:10.1200/JCO.2017.77.4794.

8. Peters S, Camidge DR, Shaw AT, et al. Alectinib versus Crizotinib in Untreated ALK-Positive Non-Small-Cell Lung Cancer. *N Engl J Med*. 2017;377(9):829-838. doi:10.1056/NEJMoa1704795.

9. Camidge DR, Dziadziuszko R, Peters S, et al. Updated Efficacy and Safety Data and Impact of the EML4-ALK Fusion Variant on the Efficacy of Alectinib in Untreated ALK-Positive Advanced Non-Small Cell Lung Cancer in the Global Phase III ALEX Study. *J Thorac Oncol*. 2019;14(7):1233-1243. doi:10.1016/j.jtho.2019.03.007.

10. Mok T, Camidge DR, Gadgeel SM, et al. Updated overall survival and final progression-free survival data for patients with treatment-naive advanced ALK-positive non-small-cell lung cancer in the ALEX study. *Ann Oncol*. 2020;31(8):1056-1064. doi:10.1016/j.annonc.2020.04.478.

11. Wu Y-L, Lu S, Lu Y, et al. Results of PROFILE 1029, a Phase III Comparison of First-Line Crizotinib versus Chemotherapy in East Asian Patients with ALK-Positive Advanced Non-Small Cell Lung Cancer. *J Thorac Oncol*. 2018;13(10):1539-1548. doi:10.1016/j.jtho.2018.06.012.

12. Zhou C, Kim S-W, Reungwetwattana T, et al. Alectinib versus crizotinib in untreated Asian patients with anaplastic lymphoma kinase-positive non-small-cell lung cancer (ALESIA): a randomised phase 3 study. *Lancet Respir Med*. 2019;7(5):437-446. doi:10.1016/S2213-2600(19)30053-0.

13. Shaw AT, Ou S-HI, Bang Y-J, et al. Crizotinib in ROS1-rearranged non-small-cell lung cancer. *N Engl J Med*. 2014;371(21):1963-1971. doi:10.1056/NEJMoa1406766.

14. Shaw AT, Riely GJ, Bang Y-J, et al. Crizotinib in ROS1-rearranged advanced non-small-cell lung cancer (NSCLC): updated results, including overall survival, from PROFILE 1001. *Ann Oncol*. 2019;30(7):1121-1126. doi:10.1093/annonc/mdz131.

15. Planchard D, Smit EF, Groen HJM, et al. Dabrafenib plus trametinib in patients with previously untreated BRAF(V600E)-mutant metastatic non-small-cell lung cancer: an open-label, phase 2 trial. *Lancet Oncol*. 2017;18(10):1307-1316. doi:10.1016/S1470-2045(17)30679-4.

16. Reck M, Rodríguez-Abreu D, Robinson AG, et al. Pembrolizumab versus Chemotherapy for PD-L1-Positive Non-Small-Cell Lung Cancer. *N Engl J Med*. 2016;375(19):1823-1833. doi:10.1056/NEJMoa1606774.

17. Reck M, Rodríguez-Abreu D, Robinson AG, et al. Updated Analysis of KEYNOTE-024: Pembrolizumab Versus Platinum-Based Chemotherapy for Advanced Non-Small-Cell Lung Cancer With PD-L1 Tumor Proportion Score of 50% or Greater. *J Clin Oncol*. 2019;37(7):537-546. doi:10.1200/JCO.18.00149.

18. Mok TSK, Wu Y-L, Kudaba I, et al. Pembrolizumab versus chemotherapy for previously untreated, PD-L1-expressing, locally advanced or metastatic non-small-cell lung cancer (KEYNOTE-042): a randomised, open-label, controlled, phase 3 trial. *The Lancet*. 2019;393(10183):1819-1830. doi:10.1016/S0140-6736(18)32409-7.

19. Gadgeel S, Rodríguez-Abreu D, Speranza G, et al. Updated Analysis From KEYNOTE-189: Pembrolizumab or Placebo Plus Pemetrexed and Platinum for Previously Untreated Metastatic Nonsquamous Non-Small-Cell Lung Cancer. *J Clin Oncol*. 2020;38(14):1505-1517. doi:10.1200/JCO.19.03136.

20. Gandhi L, Rodríguez-Abreu D, Gadgeel S, et al. Pembrolizumab plus Chemotherapy in Metastatic Non-Small-Cell Lung Cancer. *N Engl J Med*. 2018;378(22):2078-2092. doi:10.1056/NEJMoa1801005.

21. Mok TS, Wu Y-L, Ahn M-J, et al. Osimertinib or Platinum-Pemetrexed in EGFR T790M-Positive Lung Cancer. *N Engl J Med*. 2017;376(7):629-640. doi:10.1056/NEJMoa1612674.

22. Novello S, Mazieres J, Oh I-J, et al. Alectinib versus chemotherapy in crizotinib-pretreated anaplastic lymphoma kinase (ALK)-positive non-small-cell lung cancer: results from the phase III ALUR study. *Ann Oncol*. 2018;29(6):1409-1416. doi:10.1093/annonc/mdy121.

23. Solomon BJ, Besse B, Bauer TM, et al. Lorlatinib in patients with ALK-positive non-small-cell lung cancer: results from a global phase 2 study. *Lancet Oncol*. 2018;19(12):1654-1667. doi:10.1016/S1470-2045(18)30649-1.

24. Reck M, Mok TSK, Nishio M, et al. Atezolizumab plus bevacizumab and chemotherapy in non-small-cell lung cancer (IMpower150): key subgroup analyses of patients with EGFR mutations or baseline liver metastases in a randomised, open-label phase 3 trial. *Lancet Respir Med*. 2019;7(5):387-401. doi:10.1016/S2213-2600(19)30084-0.

25. Socinski MA, Jotte RM, Cappuzzo F, et al. Atezolizumab for First-Line Treatment of Metastatic Nonsquamous NSCLC. *N Engl J Med*. 2018;378(24):2288-2301. doi:10.1056/NEJMoa1716948.

26. Gottfried M, Bennouna J, Bondarenko I, et al. Efficacy and Safety of Nintedanib Plus Docetaxel in Patients with Advanced Lung Adenocarcinoma: Complementary and Exploratory Analyses of the Phase III LUME-Lung 1 Study. *Target Oncol*. 2017;12(4):475-485. doi:10.1007/s11523-017-0517-2.

27. Reck M, Kaiser R, Mellemgaard A, et al. Docetaxel plus nintedanib versus docetaxel plus placebo in patients with previously treated non-small-cell lung cancer (LUME-Lung 1): a phase 3, double-blind, randomised controlled trial. *Lancet Oncol*. 2014;15(2):143-155. doi:10.1016/S1470-2045(13)70586-2.

28. Herbst RS, Baas P, Kim D-W, et al. Pembrolizumab versus docetaxel for previously treated, PD-L1-positive, advanced non-small-cell lung cancer (KEYNOTE-010): a randomised controlled trial. *The Lancet*. 2016;387(10027):1540-1550. doi:10.1016/S0140-6736(15)01281-7.

29. Herbst RS, Garon EB, Kim D-W, et al. Long-Term Outcomes and Retreatment Among Patients With Previously Treated, Programmed Death-Ligand 1‒Positive, Advanced Non‒Small-Cell Lung Cancer in the KEYNOTE-010 Study. *JCO*. 2020;38(14):1580-1590. doi:10.1200/JCO.19.02446.

30. Nafees B, Stafford M, Gavriel S, Bhalla S, Watkins J. Health state utilities for non small cell lung cancer. *Health Qual Life Outcomes*. 2008;6(84):o. S. doi:10.1186/1477-7525-6-84.

31. Nafees B, Lloyd AJ, Dewilde S, Rajan N, Lorenzo M. Health state utilities in non-small cell lung cancer: An international study. *Asia Pac J Clin Oncol*. 2017;13(5):e195-e203. doi:10.1111/ajco.12477.

32. Chouaid C, Agulnik J, Goker E, et al. Health-related quality of life and utility in patients with advanced non-small-cell lung cancer: a prospective cross-sectional patient survey in a real-world setting. *J Thorac Oncol*. 2013;8(8):997-1003. doi:10.1097/JTO.0b013e318299243b.

33. Handorf EA, McElligott S, Vachani A, et al. Cost effectiveness of personalized therapy for first-line treatment of stage IV and recurrent incurable adenocarcinoma of the lung. *J Oncol Pract*. 2012;8(5):267-274. doi:10.1200/JOP.2011.000502.

34. Statistisches Bundesamt. Mikrozensus - Fragen zur Gesundheit: - Körpermaße der Bevölkerung 2017. https://www.destatis.de/DE/Themen/Gesellschaft-Umwelt/Gesundheit/Gesundheitszustand-Relevantes-Verhalten/Publikationen/Downloads-Gesundheitszustand/koerpermasse-5239003179004.pdf?__blob=publicationFile. Accessed February 4, 2022.

35. Kassenärztliche Bundesvereinigung. *Online Version des EBM*; 2020. https://www.kbv.de/html/online-ebm.php. Accessed June 30, 2020.

36. AIO, iOMedico. *CRISP Register Platform Lung Cancer (AIO-TRK-0315): Special Analysis Database cut 30.06.2020*. (unpublished data); 2021.

37. Lung Cancer Group Cologne. Treibermutationen. https://lungcancergroup.de/molekularpathologie/treibermutationen/. Accessed September 9, 2020.

38. Cross DAE, Ashton SE, Ghiorghiu S, et al. AZD9291, an irreversible EGFR TKI, overcomes T790M-mediated resistance to EGFR inhibitors in lung cancer. *Cancer Discov*. 2014;4(9):1046-1061. doi:10.1158/2159-8290.CD-14-0337.

39. Oxnard GR, Arcila ME, Sima CS, et al. Acquired resistance to EGFR tyrosine kinase inhibitors in EGFR-mutant lung cancer: distinct natural history of patients with tumors harboring the T790M mutation. *Clin Cancer Res*. 2011;17(6):1616-1622. doi:10.1158/1078-0432.CCR-10-2692.

40. Sequist LV, Waltman BA, Dias-Santagata D, et al. Genotypic and histological evolution of lung cancers acquiring resistance to EGFR inhibitors. *Sci Transl Med*. 2011;3(75):75ra26. doi:10.1126/scitranslmed.3002003.

41. Hata A, Katakami N, Yoshioka H, et al. Rebiopsy of non-small cell lung cancer patients with acquired resistance to epidermal growth factor receptor-tyrosine kinase inhibitor: Comparison between T790M mutation-positive and mutation-negative populations. *Cancer*. 2013;119(24):4325-4332. doi:10.1002/cncr.28364.

42. Yu HA, Arcila ME, Rekhtman N, et al. Analysis of tumor specimens at the time of acquired resistance to EGFR-TKI therapy in 155 patients with EGFR-mutant lung cancers. *Clin Cancer Res*. 2013;19(8):2240-2247. doi:10.1158/1078-0432.CCR-12-2246.

43. Arcila ME, Oxnard GR, Nafa K, et al. Rebiopsy of lung cancer patients with acquired resistance to EGFR inhibitors and enhanced detection of the T790M mutation using a locked nucleic acid-based assay. *Clin Cancer Res*. 2011;17(5):1169-1180. doi:10.1158/1078-0432.CCR-10-2277.

44. Douillard J-Y, Ostoros G, Cobo M, et al. Gefitinib Treatment in EGFR Mutated Caucasian NSCLC: Circulating-Free Tumor DNA as a Surrogate for Determination of EGFR Status. *J Thorac Oncol*. 2014;9(9):1345-1353. doi:10.1097/JTO.0000000000000263.

45. Vanderlaan PA, Yamaguchi N, Folch E, et al. Success and failure rates of tumor genotyping techniques in routine pathological samples with non-small-cell lung cancer. *Lung Cancer*. 2014;84(1):39-44. doi:10.1016/j.lungcan.2014.01.013.

46. Hagemann IS, Devarakonda S, Lockwood CM, et al. Clinical next-generation sequencing in patients with non-small cell lung cancer. *Cancer*. 2015;121(4):631-639. doi:10.1002/cncr.29089.

47. Chouaid C, Dujon C, Do P, et al. Feasibility and clinical impact of re-biopsy in advanced non small-cell lung cancer: a prospective multicenter study in a real-world setting (GFPC study 12-01). *Lung Cancer*. 2014;86(2):170-173. doi:10.1016/j.lungcan.2014.08.016.

48. Remon J, Lacroix L, Jovelet C, et al. Real-World Utility of an Amplicon-Based Next-Generation Sequencing Liquid Biopsy for Broad Molecular Profiling in Patients With Advanced Non-Small-Cell Lung Cancer. *JCO Precis Oncol*. 2019;3. doi:10.1200/PO.18.00211.

49. Clark TA, Chung JH, Kennedy M, et al. Analytical Validation of a Hybrid Capture-Based Next-Generation Sequencing Clinical Assay for Genomic Profiling of Cell-Free Circulating Tumor DNA. *J Mol Diagn*. 2018;20(5):686-702. doi:10.1016/j.jmoldx.2018.05.004.

50. Foundation Medicine. *FoundationOne CDx™ Technische Spezifikationen*; 2020. https://www.foundationmedicine.at/content/dam/rfm/at_v2-de_at/F1LCDx/Genliste_CDx.pdf. Accessed April 6, 2021.
